# Hydrogen peroxide sensing, signaling and regulation of transcription factors

**DOI:** 10.1016/j.redox.2014.02.006

**Published:** 2014-02-23

**Authors:** H. Susana Marinho, Carla Real, Luísa Cyrne, Helena Soares, Fernando Antunes

**Affiliations:** a Departamento de Química e Bioquímica, Centro de Química e Bioquímica, Faculdade de Ciências, Universidade de Lisboa, Lisboa, Portugal; b Instituto Gulbenkian de Ciência, Oeiras, Portugal; c Escola Superior de Tecnologia da Saúde de Lisboa, IPL, Lisboa, Portugal

**Keywords:** Redox signaling, Localized H_2_O_2_ concentrations, Rate constants, Thiol reactivity, Cytosol-nuclear traffic, DNA binding and transactivation, AD, activation domain, ER, endoplasmic reticulum, GPx, glutathione peroxidases, NES, nuclear exporting signal, NLS, nuclear localization signal, PHD, prolyl hydroxylase, Prxs, peroxiredoxins, TF, transcription factor, Ub, Ubiquitin

## Abstract

The regulatory mechanisms by which hydrogen peroxide (H_2_O_2_) modulates the activity of transcription factors in bacteria (OxyR and PerR), lower eukaryotes (Yap1, Maf1, Hsf1 and Msn2/4) and mammalian cells (AP-1, NRF2, CREB, HSF1, HIF-1, TP53, NF-κB, NOTCH, SP1 and SCREB-1) are reviewed. The complexity of regulatory networks increases throughout the phylogenetic tree, reaching a high level of complexity in mammalians. Multiple H_2_O_2_ sensors and pathways are triggered converging in the regulation of transcription factors at several levels: (1) synthesis of the transcription factor by upregulating transcription or increasing both mRNA stability and translation; (ii) stability of the transcription factor by decreasing its association with the ubiquitin E3 ligase complex or by inhibiting this complex; (iii) cytoplasm–nuclear traffic by exposing/masking nuclear localization signals, or by releasing the transcription factor from partners or from membrane anchors; and (iv) DNA binding and nuclear transactivation by modulating transcription factor affinity towards DNA, co-activators or repressors, and by targeting specific regions of chromatin to activate individual genes. We also discuss how H_2_O_2_ biological specificity results from diverse thiol protein sensors, with different reactivity of their sulfhydryl groups towards H_2_O_2_, being activated by different concentrations and times of exposure to H_2_O_2_. The specific regulation of local H_2_O_2_ concentrations is also crucial and results from H_2_O_2_ localized production and removal controlled by signals. Finally, we formulate equations to extract from typical experiments quantitative data concerning H_2_O_2_ reactivity with sensor molecules. Rate constants of 140 M^−1^ s^−1^ and ≥1.3 × 10^3^ M^−1^ s^−1^ were estimated, respectively, for the reaction of H_2_O_2_ with KEAP1 and with an unknown target that mediates NRF2 protein synthesis. In conclusion, the multitude of H_2_O_2_ targets and mechanisms provides an opportunity for highly specific effects on gene regulation that depend on the cell type and on signals received from the cellular microenvironment.

## Highlights

• Complexity of redox regulation increases along the phylogenetic tree.
• Complex regulatory networks allow for a high degree of H_2_O_2_ biological plasticity.
• H_2_O_2_ modulates gene expression at all steps from transcription to protein synthesis.
• Fast response (s) is mediated by sensors with high H_2_O_2_ reactivity.
• Low reactivity H_2_O_2_ sensors may mediate slow (h) or localized H_2_O_2_ responses.

## Graphical Abstract

**Figure:**
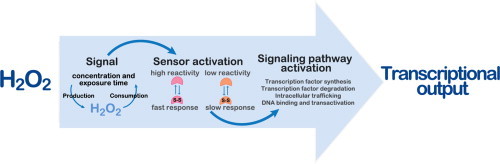

## Introduction

Hydrogen peroxide (H_2_O_2_) is a ubiquitous oxidant present in all aerobic organisms. Since its first identification in a living cell, H_2_O_2_ was considered a toxic byproduct of aerobic metabolism, something that cells had to remove [1]. If H_2_O_2_ detoxification catalyzed by catalases and peroxidases was not adequate, H_2_O_2_ would diffuse and oxidize biological targets causing cellular malfunctions responsible for several pathologies and aging. Favoring this paradigm was the discovery that neutrophils use H_2_O_2_ toxicity and produce massive amounts of H_2_O_2_ during the oxidative burst to kill invading pathogens. In the 70s some isolated observations already supported a role for H_2_O_2_ as a signaling molecule, *e.g.* H_2_O_2_ was found to mimic insulin action [2] or to activate guanylate cyclase [3]. Apparently, these observations remained mostly unnoticed in the field of oxidative stress, but at the end of the 80s some key discoveries built up on them. In 1987, it was found that H_2_O_2_ at micromolar levels elicits arterial pulmonary relaxation mediated by the activation of guanylate cyclase [4] and in 1989, H_2_O_2_ was found to potentiate tyrosine phosphorylation during insulin signaling [5] and to stimulate cell proliferation at low concentrations [6]. Also in 1989, OxyR was identified as the transcription factor (TF) targeted by H_2_O_2_ in the adaptive response of *Escherichia Coli* (*E. coli*) and *Salmonella typhymurium* (*S. typhymurium*) [7], and in 1990 NF-κB was identified as a redox regulated TF [8]. In the following year, the activation of NF-κB by H_2_O_2_ was discovered in a publication [9] that had a profound impact in the field, with near 3500 citations so far. Also in 1991, NADPH oxidases were identified in non-phagocytic cells as H_2_O_2_ producing systems [10,11]. If H_2_O_2_ was a toxic species, why were cells intentionally producing this species by a complex regulated mechanism? Concomitantly, several clinical trials based on the notion that oxidants were toxic and antioxidants were beneficial for cancer prevention were largely unsuccessful as reviewed in [12]. Nowadays, redox biology is an established field and the essential regulating role played by H_2_O_2_ *in vivo* with important implications in health and disease is unquestionable. However, there are still a lot of unanswered questions regarding our understanding of redox-dependent regulation of gene expression. What makes a good H_2_O_2_ sensor? What are the common chemical and kinetic principles that govern H_2_O_2_ signaling? Is it possible to obtain an integrative view of H_2_O_2_ regulation of TFs?

In this review, we will start by discussing what characteristics an H_2_O_2_ sensor should have; we review the chemistry of H_2_O_2_, mainly its reaction with thiols. The aim is to give a brief overview of basic chemical and kinetic principles that govern H_2_O_2_ signaling. Next, we describe briefly the TFs reviewed here, which include bacterial (OxyR and PerR), yeast (Yap1, Msn2/4, Maf1, and Hsf1), and mammalian (AP-1, NRF2, CREB, TP53, NOTCH, NF-kB, SP1, HIF-1, SREBP-1 and HSF1) TFs. The main body of this article describes the redox regulation of these TFs by H_2_O_2_. A detailed review on each of the TFs listed is not intended, as there are many excellent reviews that do so. We aim to give an integrative review of their regulation by H_2_O_2_ at several steps: synthesis and stability of the TF, cytoplasm-nuclear trafficking and DNA binding and transactivation, so that the reader is made aware of the diversity of mechanisms by which H_2_O_2_ regulates TFs and also what the common themes in H_2_O_2_-regulated signaling pathways are.

## What makes a good sensor for H_2_O_2_?

The characteristics of a good sensing molecule for H_2_O_2_ can be derived from basic concepts taken from information theory and chemistry. Low-molecular weight thiols react slowly with H_2_O_2_, as exemplified by the rate constants for H_2_O_2_ reaction with cysteine and reduced glutathione (GSH), which are respectively 2.9 M^−1^ s^−1^ and 0.87 M^−1 ^s^−1^ (pH 7.4, see Table 1). The reaction of thiols with H_2_O_2_ involves a nucleophilic attack of the thiolate on H_2_O_2_ and, as such, thiol reactivity is driven by the pKa of the sulfhydryl (−SH) group. Since the pKa of the SH group in cysteine is 8.3 only about 10% of free cysteine is ionized at the physiological pH. In proteins, the electrostatic environment around the SH group of cysteine residues may render these groups more acidic and, therefore, they may have an increased reactivity towards H_2_O_2_, since a higher fraction will be in the thiolate form. Nucleophilicity is also an important factor and, in several proteins, a lower stabilization of the thiolate in cysteine residues increases nucleophilicity of the thiolate [13] and increases, by several orders of magnitude, the rate constants with H_2_O_2_ (see Table 1). The concept of redox signaling by H_2_O_2_ was proposed following the discovery of proteins involved in signaling, such as phosphatases, kinases and transcription factors, that contain cysteine residues whose SH groups are oxidized (Fig. 1) causing a change of their biological activity. According to this paradigm upon an increase in the concentration of H_2_O_2_, these proteins are specifically oxidized, and a cascade of molecular events ensues. Unfortunately, the wealth of data identifying reactive SH groups, *i.e.* groups that are oxidized upon exposure to an oxidant, contrasts with the near absence of quantitative kinetic data characterizing this reactivity. The few rate constants listed in Table 1 show that the reactivity with H_2_O_2_ of signaling proteins like the phosphatases Cdc25B and PTB1B is much lower than the reactivity of peroxiredoxins (Prxs), of the selenocysteine residues present in glutathione peroxidases (GPx), or of the heme center present in catalase. In addition, the cellular abundance of antioxidant enzymes like GPx, Prxs and catalase is much larger than that of signaling proteins like phosphatases or TFs. This is important since in the reaction of H_2_O_2_ with thiols we are dealing with second order rate constants, *i.e.* the rate of reaction is proportional to the concentrations of H_2_O_2_ and the thiol. The consequence is that signaling molecules cannot compete with known protein antioxidant systems that remove H_2_O_2_. In addition, existing data show that several types of GPx (at least eight isoenzymes) and Prxs (six isoenzymes) coexist [14,15]. If these enzymes had only an antioxidant function, why is there such a variety? For all these reasons, it was concluded that a signaling protein like PTP1B that is redox regulated by H_2_O_2_ [[16], [17], [18]] but has a low reactivity towards H_2_O_2_ [19], could not be a direct sensor of H_2_O_2_ [13,[20], [21], [22]]. Also, antioxidant systems like Prxs and GPx would constitute a kinetic bottleneck that avoids any significant reaction of H_2_O_2_ with signaling low-reactive proteins [13]. Instead, a high reactive protein, like a peroxiredoxin or a glutathione peroxidase, would be the initial H_2_O_2_ sensor, which through a thiol-disulfide reshuffling transfer reaction would then oxidize the target protein. This paradigm was inspired in the activation mechanism of the OxyR TF in bacteria [23]. However, these kinetic considerations do not tell the whole story.

**Fig. 2:**
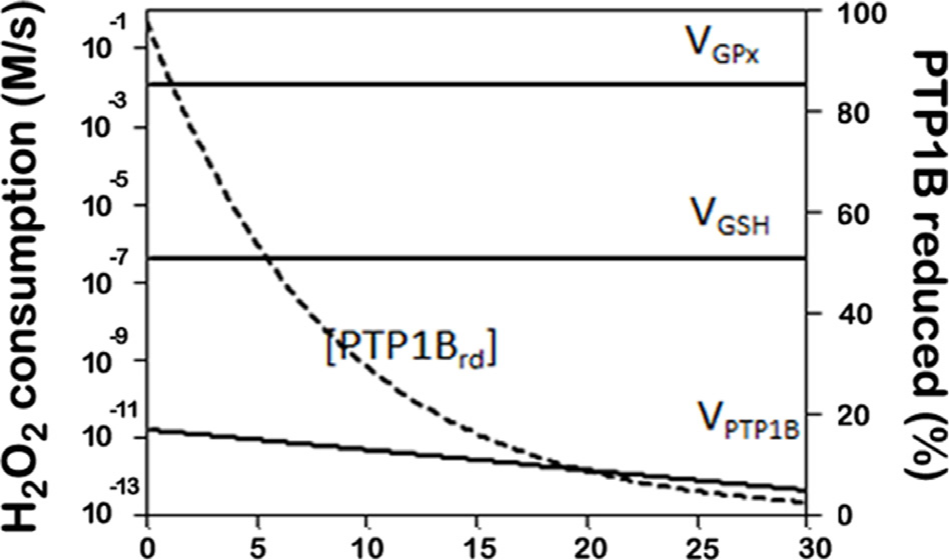
PTP1B signaling by H_2_O_2_ when in the presence of an antioxidant kinetic bottleneck that outcompetes the rate of PTP1B oxidation.The following reactions were included: a rate of H_2_O_2_ production of 1.2 × 10^−2^ M s^−1^; H_2_O_2_ consumption *via* glutathione peroxidase (*V*_GPx_ = *k*_GPx_ × [GPx] × [H_2_O_2_]), *via* PTP1B (*V*_PTP1B_ = *k*_PTP1b_× [PTP1B_rd_] × [H_2_O_2_]) and *via* non-enzymatic reaction with GSH (*V*_GSH_ = *k*_GSH_ × [GSH] × [H_2_O_2_]). *k*_GPx_ = 6 × 10^7^ M^−1^ s^−1^, [GPx] = 2 × 10^−6^ M, *k*_PTP1b_ = 20 M^−1^ s^−1^, [PTP1B_tot_] = 8.3 × 10^−9^ M, *k*_GSH_ = 0.87 M^−1 ^s^−1^, [GSH] = 5 × 10^−3^ M. With these parameters, the steady state obtained was [H_2_O_2_] = 1 × 10^−4^ M. [PTP1B_tot_] = [PTP1B_rd_] + [PTP1B_ox_], where the subscripts tot, rd and ox, refer to the total amount of PTP1B, to the reduced and to the oxidized forms of PTP1B, respectively.

(1) Different H_2_O_2_ signaling pathways are triggered by different H_2_O_2_ concentrations and occur with different kinetics. For example exposure of H4IIEC hepatocytes to extracellular H_2_O_2_ (25–50 µM) for 3 h decreased insulin-stimulated AKT phosphorylation, and increased the phosphorylation of both JNK and its substrate c-JUN, while lower concentrations of H_2_O_2_ (5–10 µM) enhanced insulin-stimulated phosphorylation of AKT [24]. In addition, H_2_O_2_ exerts often biphasic responses in which one effect is reversed in a narrow range of concentration such as in H_2_O_2_ regulation of fatty acid synthase [[25], [26], [27]]. If the initial target is a high-reactive molecule, it is hard to imagine such quantitative diversity in H_2_O_2_ response.
(2) More importantly, information is not mass. That antioxidant systems impose a kinetic bottle-neck for the flux of H_2_O_2_, and that a rate of oxidation of a sensor is vastly outcompeted by the rate of oxidation of antioxidant systems is irrelevant for a sensing mechanism. In Fig. 2, we simulate a situation where an antioxidant system outcompetes the reaction of H_2_O_2_ with PTP1B by nine orders of magnitude and, in spite of that, PTP1B is oxidized with a half-life of 5.7 min, a time scale typical of a signaling response. The role of a sensor is to interact selectively with the signaling molecule and to produce an effect that can be measured by a transducer. So, its main role is to transmit information and not, *e.g.* to be a bulk catalyst in a biochemical pathway. What is important is that a variation of H_2_O_2_ concentration is sensed and this information is transmitted downstream the signaling cascade. By sensing we mean the rate of oxidation of the sensor increases/decreases upon an increase/decrease in the H_2_O_2_ concentration (the signal). If the rate of oxidation of the sensor is many orders of magnitude lower than the rate of production of H_2_O_2_ or the rate of H_2_O_2_ consumption by antioxidant systems, this is actually a good characteristic for a sensor. An ideal sensor does not change the intensity of the signal, it just responds to a change in the signal. For example, a thermometer in a water bath senses changes in the temperature, and does not decrease or increase the temperature of the water. One biochemical illustration of this is the HIF system sensing O_2_. In this system, a prolyl hydroxylase (PHD) catalyzes the hydroxylation of the subunit HIF-1α by O_2_, which is then subsequently marked for degradation [28]. The fraction of O_2_ consumed by PHD compared with the overall cellular O_2_ consumption is small, but this does not prevent it from being an O_2_ sensor.

**Table 1:** Rate constants for the reactions between H_2_O_2_ and several thiol and metal proteins. The intracellular steady-state H_2_O_2_ concentrations needed to obtain a response time of 30 s, 5 min and 1 h were calculated with Eq. (7).

	Rate constant (M^−1^ s^−1^)	[H_2_O_2_] needed for a response time of 30 s (µM)	[H_2_O_2_] needed for a response time of 5 min (µM)	[H_2_O_2_] needed for a response time of 1 h (µM)
Thiol-protein				
GSH	0.87	2.7 × 10^4^	2.7 × 10^3^	220
Thioredoxin	1.05	2.2 × 10^4^	2.2 × 10^3^	180
PTP1B	20	1.2 × 10^3^	120	9.6
KEAP1	140	170	17	1.4
Cdc25B	160	140	14	1.2
GAPDH	500	46	4.6	0.39
Peroxiredoxin-5	3.0 × 10^5^	0.077	7.7 × 10^−3^	6.4 × 10^−4^
Peroxiredoxin-2	1.0 × 10^7^	2.3 × 10^−3^	2.3 × 10^−4^	1.9 × 10^−5^

Metal-protein				
PerR	1.0 × 10^5^	0.23	2.3 × 10^−2^	1.9 × 10^−3^
Catalase	2.0 × 10^7^	1.2 × 10^−3^	1.2 × 10^−4^	9.6 × 10^−6^

The rate constant for the reaction of H_2_O_2_ with KEAP1, was estimated in this work from data from [189]. The other rate constants are for pH 7.4−7.6 at 37 °C unless noted otherwise. References: GSH [361] GAPDH [362] estimated for 37 °C from 100 M^−1^s^−1^ measured at 0 °C; thioredoxin [363]; PTP1B [19]; Cdc25B [364]; human peroxiredoxin 2 [365] and human peroxiredoxin 5 [366] both at 20–25 °C; PerR [300] at pH 7; and catalase [367].

**Fig. 1:**
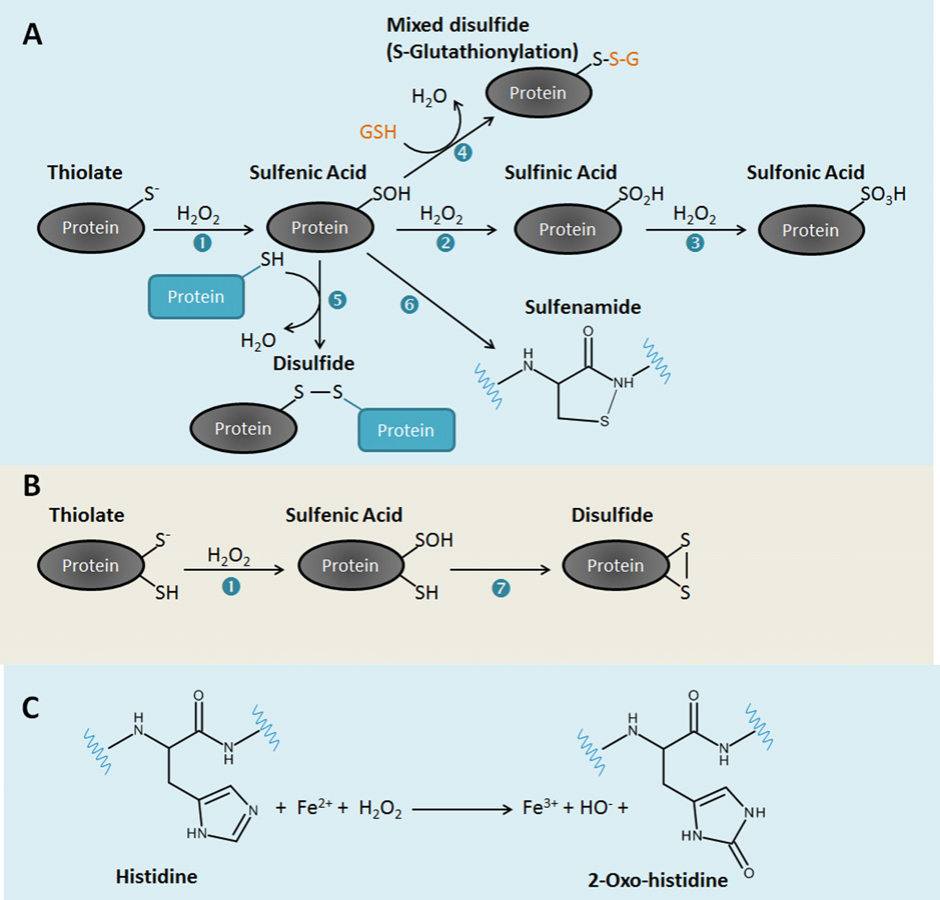
Oxidative modifications of cysteine **(A** and **B)** and histidine **(C)** residues in proteins induced by H_2_O_2_. In cells, sulfhydryl (SH) groups of cysteine residues with low pKa may ionize forming thiolates. Thiolates are good nucleophiles and form a sulfenic acid (SOH) upon reaction with H_2_O_2_ (reaction 1). Once formed, the SOH can be reduced to a disulfide by a reaction with the SH group of another cysteine residue either in the same (reaction 7) or in a second protein (reaction 5). Alternatively, a SOH can react with the low molecular weight thiol glutathione (GSH) (reaction 4) to form a mixed disulfide in a reaction known as *S*-glutathionylation or *S*-thiolation. In an event where a neighboring cysteine residue or GSH is absent, the amide nitrogen of a neighboring amino acid residue can attack the SOH to form a sulfenamide (reaction 6). This reaction occurs in PTP1B. The SOH can also react further with H_2_O_2_ to generate more oxidized forms of sulfur, the sulfinic acid (SO_2_H) (reaction 2) and sulfonic acid SO_3_H (reaction 3). Disulfides can be reduced back to thiols using the thioredoxin/thioredoxin reductase and glutaredoxin/GSH/glutathione reductase systems. Sulfinic acids in 2-cys Prxs, but not other proteins, can be reduced to thiols using the enzyme sulfiredoxin [372]. No known enzyme is able to catalyze the reduction of sulfonic acids in proteins. In proteins containing iron metal centers such as PerR, histidine residues can be oxidized by H_2_O_2_ in a Fenton-like reaction possibly involving the formation of the hydroxyl radical as an intermediate, to form 2-oxo-histidine.

Thus, a putative target with reactivity towards H_2_O_2_ much lower than other molecules also present in the system does not, *per se*, exclude it from being a sensor. Next, we evaluate whether the known characteristics of low-reactivity thiol proteins are compatible with a role as H_2_O_2_ sensors. A chemical sensor should have the following characteristics.

(1) It does not consume the chemical signal it is responding to, which, as we have seen, is verified for low-reactivity thiol proteins. Sensor functions may be combined with other functions, as is the case of peroxiredoxin 1 in the AKT signaling pathway, in which H_2_O_2_ sensing and control of H_2_O_2_ are combined in the same molecule [29].
(2) It should be sensitive to changes in the concentration of the chemical signal it is sensing. Visiting again O_2_ sensing by the HIF system, the *K*_m_ towards O_2_ of PHD is 100 μM [30], much higher than the endogenous concentration of O_2_ (approximately 30 μM), and so the HIF system responds to O_2_ changes in the operational O_2_ concentration range *in vivo*. When the O_2_ concentration falls, the rate of hydroxylation of HIF-1α decreases and, consequently, HIF is not degraded and triggers gene expression. In the case of protein thiols, the reaction between the thiol and H_2_O_2_ is a second-order reaction, and so the rate of reaction depends on H_2_O_2_ concentration.
(3) Finally, a sensor should have dynamic characteristics that suit its function. The reactivity of the sensor has to be such that before the H_2_O_2_ signal is terminated the sensor is activated, *i.e.* it is oxidized by H_2_O_2_. To analyze this issue the reactivity of thiol proteins towards H_2_O_2_ needs to be evaluated.

To help this analysis, a minimal mathematical model can be set up according to the following two reactions:

(1) $${\text{Target}}_{\text{reduced}}+{\text{H}}_{2}{\text{O}}_{2}\to {\text{Target}}_{\text{oxidized}}+{\text{H}}_{2}\text{O}+\frac{1}{2}\;{\text{O}}_{2}$$

(2) $${\text{Target}}_{\text{oxidized}}\to {\text{Target}}_{\text{reduced}}$$

For these two reactions the rate laws are defined as follows:

• For the activation step (1) *v*_1_ = *k*_activation_ × [Target_reduced_], where *k*_activation_ = *k*_target + H_2_O_2__ × [H_2_O_2_]. *k*_target + H_2_O_2__ is the rate constant for the direct reaction between H_2_O_2_ and the thiol protein.
• For the switch-off step (2), in which the oxidized protein is regenerated back to the reduced form, *v*_2_ = *k*_switchoff_ × [Target_oxidized_] = *k*_switchoff_ × ([Target]_total_ − [Target_reduced_]), assuming that the total concentration of the target protein is constant ([Target]_total_ = [Target_oxidized_] + [Target_reduced_]).

With this, the following differential equation is set up, where Target_reduced_ is the fraction of the target thiol protein in the reduced state:

(3) $$\frac{d{\text{Target}}_{\text{reduced}}}{dt}=k_{\text{switch}\;\text{off}}(1-{\text{Target}}_{\text{reduced}})-k_{activation}{\text{Target}}_{\text{reduced}}$$

The analytical solution of Eq. (3) is the following:

(4) $$\begin{array}{c} {\text{Target}}_{\text{reduced}}{|}_{t}=\frac{k_{\text{switch}\;\text{off}}}{k_{\text{switch}\;\text{off}}+k_{\text{activation}}}+e^{-(k_{\text{switch}\;\text{off}}+k_{\text{activation}})t}\times \\ \times ({\text{Target}}_{\text{reduced}}{|}_{0}-\frac{k_{\text{switch}\;\text{off}}}{k_{\text{switch}\;\text{off}}+k_{\text{activation}}}) \end{array}$$

(5) $${\text{Target}}_{\text{reduced}}{|}_{t}=1-{\text{Target}}_{\text{oxidized}}{|}_{t}$$

Some useful information can be taken from (4), (5)):

• If the activation of the thiol protein is not switched-off (*i.e.*, *k*_switchoff_ = 0), Eq. (4) simplifies to a simple exponential decay (Eq. (6)) and the response time, defined as half of the total response, is given by Eq. (7):

(6) $$\begin{array}{l} {\text{Target}}_{\text{reduced}}{|}_{t}=e^{-k_{\text{activation}}t}({\text{Target}}_{\text{reduced}}{|}_{0})\\ \Leftrightarrow \ln(\frac{{\text{Target}}_{\text{reduced}}{|}_{t}}{{\text{Target}}_{\text{reduced}}{|}_{0}})=-k_{\text{activation}}\times t \end{array}$$

(7) $$\tau_{1/2}=\frac{\ln(2)}{k_{\text{activation}}}=\frac{\ln(2)}{k_{\text{target}+{\text{H}}_{2}{\text{O}}_{2}}\times [{\text{H}}_{2}{\text{O}}_{2}]}$$

Eq. (7) can be manipulated to calculate not only the *τ*_1/2_, but also the [H_2_O_2_] or the *k*_target + H_2_O_2__, provided two of these parameters are known. In this case, at the end of the response all protein will be activated since there is not an operating switch-off mechanism.

• If the thiol protein is switched-off (*k*_switchoff_ > 0), the steady-state fraction of protein present in the oxidized form is given by Eq. (8) and the response time, defined as half of the total response, is given by Eq. (9):

(8) $${\text{Target}}_{\text{oxidized}}{|}_{\text{steadystate}}=1-\frac{k_{\text{switch}\;\text{off}}}{k_{\text{switch}\;\text{off}}+k_{\text{activation}}}$$

(9) $$\tau_{1/2}=\frac{\ln(2)}{k_{\text{activation}}+k_{\text{switch}\;\text{off}}}$$

**Fig. 3:**
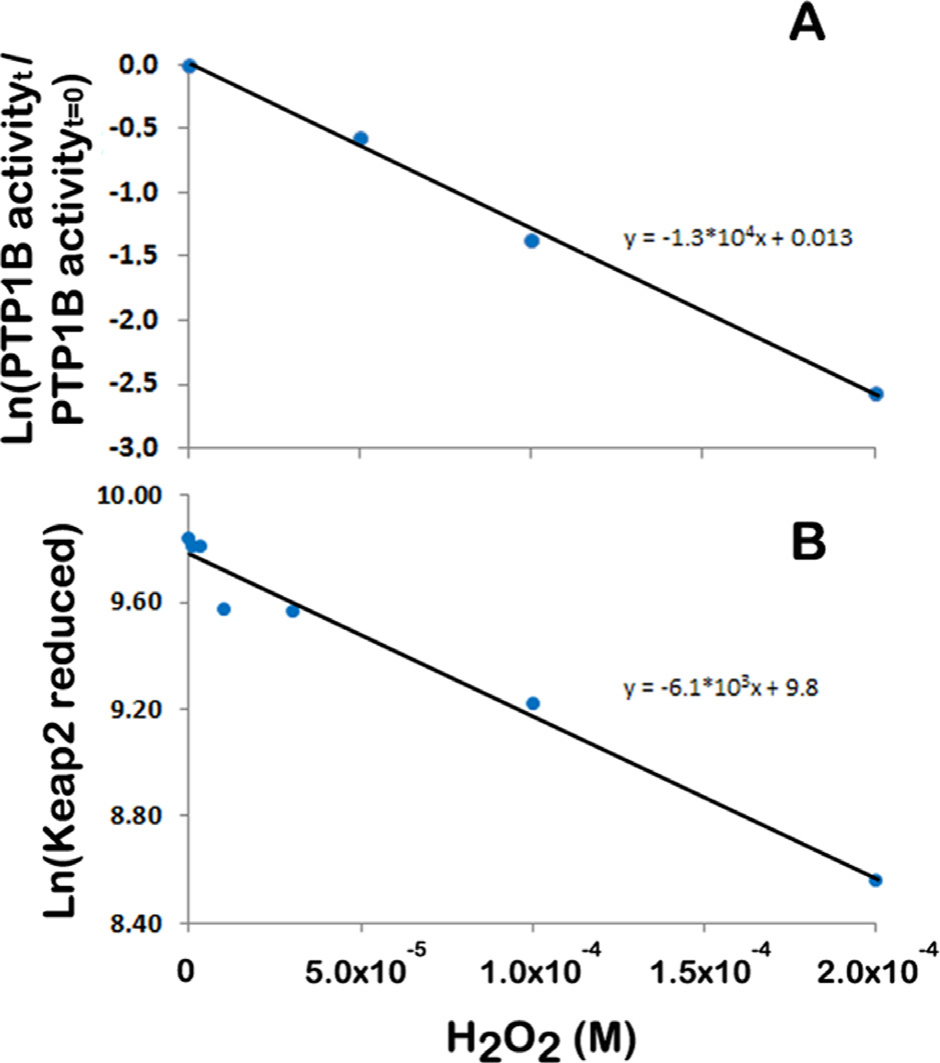
Application of Eq. (6) to estimate rate constants between cellular targets and H_2_O_2_. Plot of the fraction of PTP1B activity observed *invitro* after 10 min **(A)** and of the reduced form of KEAP1 observed in HeLa cells after 5 min **(B)** of incubation with the indicated H_2_O_2_ concentrations. Experimental data are taken from [33] and [189], respectively for PTP1B and KEAP1. If a simple exponential decay is considered, that is no regeneration of sensor occurs, the slope of these plots is *k*_target + H_2_O_2__ × *t* (Eq. (6)) and, therefore, the rate constants between PTP1B and KEAP1 with H_2_O_2_ are estimated at 22 M^−1^ s^−1^ and 20 M^−1 ^s^−1^, respectively. If a gradient between extracellular and intracellular H_2_O_2_ of 6.8 is considered in HeLa cells [42], the rate constant for H_2_O_2_ reaction with KEAP1 is estimated at 140 M^−1^ s^−1^.

In this article we will apply only (6), (7) because there are no quantitative data available for the switch-off mechanism that regenerates the H_2_O_2_ sensor. This approximation is acceptable for time courses where the switch-off mechanisms are not operating at a significant rate. In Table 1, we show H_2_O_2_ concentrations needed to have a response time of 30 s, 5 min and 1 h, calculated by applying Eq. (7) for those proteins whose rate constant with H_2_O_2_ is known. In other words, the intracellular steady-state H_2_O_2_ concentrations indicated are those necessary to oxidize the listed proteins by 50% after exposing it to H_2_O_2_ for 30 s, 5 min and 1 h. As can be observed, if a fast response is necessary (30 s), or if the H_2_O_2_ transient signal lasts only 30 s, only Prxs, PerR and catalase are sufficiently sensitive targets as to provide the desired response. For other targets, like PTP1B, the H_2_O_2_ signaling concentration needed to trigger the response during the 30 s of the duration of the signal would be too high, 1.2 mM. However, if cells require a slow response (1 h), or if the H_2_O_2_ transient signal lasts for 1 h, even a low reactive sensor, such as Cdc25B, will be sufficient to mediate the signaling pathway, as exposure to a 1.2 μM H_2_O_2_ concentration during 1 h would be enough to activate the response, *i.e.* to oxidize Cdc25B by 50%. Thus, the duration of the transient H_2_O_2_ signal is an important experimental observation that gives a hint on whether a sensor with high or low reactivity is operating. In this regard, for example, H_2_O_2_ production triggered by EGF peaks at 5 min, and returns to baseline after 20 min [31] or 60 min [32]. While a short H_2_O_2_ transient signal excludes the possibility that a low-reactive-sensor is operating, a long transient signal is compatible with both a high and low-reactivity sensor. The same kind of information can be inferred from the time course of the signaling pathway: a very fast response is incompatible with a low-reactive sensor, while a slow response may be the result of either a low-reactive sensor that takes time to respond or, alternatively, the result of a high-reactive sensor that responds rapidly, but then oxidizes slowly an effector molecule. Using PTP1B as an example to analyze these two scenarios, analysis of experimental data where recombinant PTP1B inactivation was studied as a function of H_2_O_2_ concentration *in vitro* [33] revealed a *k*_target + H_2_O_2__ = 22 M^−1^ s^−1^ (Fig. 3), which is near the published values (see Table 1) establishing this protein as a low reactivity thiol sensor protein. Also, in the same work, *in vivo* activation by EGF caused a 35% inactivation of PTP1B after 5 min. If a direct oxidation of PTP1B by H_2_O_2_ with a rate constant *k*_target + H_2_O_2__ = 22 M^−1^ s^−1^ is assumed, a local concentration of H_2_O_2_ near 66 µM would be needed. However, if we consider that a high-reactive thiol sensor protein reacts with H_2_O_2_, and then relays the signal to PTP1B, a lower H_2_O_2_ local concentration would be needed. An extra layer of uncertainty is whether the rate constants determined *in vitro* are the same operating for the reaction *in vivo* and whether H_2_O_2_ derivatives, like peroxymonophosphate [34] and peroxymonocarbonate [35,36], which have higher reactivity towards PTP1B, operate *in vivo*.

Another important parameter to take into account when discussing sensors is the intensity of the H_2_O_2_ signal, and hence the notion of localized H_2_O_2_ concentrations should also be considered. The extracellular H_2_O_2_ threshold concentration that triggers apoptosis in Jurkat T-cells is 7 µM [37], which considering the H_2_O_2_ gradient across the plasma membrane, converts to an intracellular H_2_O_2_ concentration probably lower than 1 µM [38]. However, cells may tolerate relatively high localized H_2_O_2_ concentrations for a short period of time. In recent years it became clear that cells have developed several strategies to insure signaling H_2_O_2_ concentrations are reached only in localized compartments near the site of its production [39]. For example, H_2_O_2_-dependent redox regulation of PTP1B requires colocalization of PTP1B with the NADPH oxidase Nox4 in the endoplasmic reticulum, with cytosolic PTP1B being insensitive to overexpression of endoplasmic-reticulum Nox4 [40].

Upon activation of receptor activated kinases, H_2_O_2_ is produced either in specific endosomes or in localized sites near the plasma membrane depending on the cell type. Biomembranes constitute a permeability barrier to H_2_O_2_ [38,[41], [42], [43]] and may help maintain higher H_2_O_2_ concentrations near its local of production. Because the permeability of the plasma membrane is regulated by H_2_O_2_ [[44], [45], [46]], it may be hypothesized that membrane domains near the site of H_2_O_2_ production are altered in order to have a lower permeability towards H_2_O_2_. Indeed, plasma membrane permeability towards H_2_O_2_ may range from near complete permeable in yeast mutants of the ergosterol pathway [44] to a near complete impermeability in human spermatozoa [47]. Furthermore, aquaporins also regulate H_2_O_2_ transport across biomembranes [48] and mediate intracellular H_2_O_2_ signaling [49], providing an additional potential control step.

**Fig. 4:**
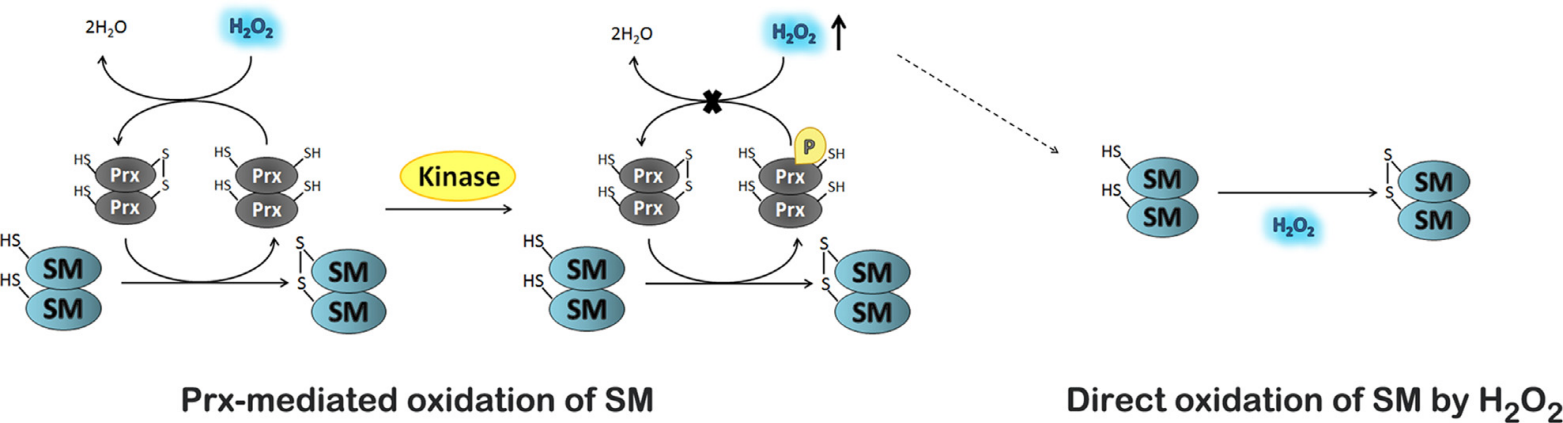
Localized increase of H_2_O_2_ levels mediated through inhibition of peroxiredoxins activity by its phosphorylation. Peroxiredoxins (Prxs) can act as highly reactive H_2_O_2_ sensors and transduce the signal to a signaling molecule (SM). Alternatively, upon binding of a ligand to a membrane receptor an SRC family kinase can be activated. This SRC kinase activates NADPH oxidase in the plasma membrane, which leads to the production of H_2_O_2_, and catalyzes phosphorylation of Prx at a tyrosine residue leading to its inactivation. Prx inactivation leads to a transient accumulation of H_2_O_2_ around membranes, where signaling components are concentrated. This will promote the direct oxidation of H_2_O_2_ sensors with intermediate and low reactivity.

In addition to a localized production of H_2_O_2_, the local inhibition of antioxidant systems, like Prxs, also contributes for a localized increase in the concentration of H_2_O_2_ [50,51]. Such strategy is reminiscent of the signaling mediated by phosphorylation in which both activation of kinases and inhibition of phosphatases occur. Concerning the inactivation of Prxs, two strategies have been proposed. (i) The so-called floodgate hypothesis in which an overoxidation of the catalytic cysteine residues of Prxs results in the inhibition of the peroxiredoxin-catalyzed reduction of H_2_O_2_. Overoxidation of peroxiredoxin is observed with a high concentration of H_2_O_2_, but recent studies showed that H_2_O_2_ levels reached during signaling are not enough to overoxidize peroxiredoxin [29,50]. (ii) An alternative strategy is the inhibition of peroxiredoxin activity by its phosphorylation [50,15] (Fig. 4). The work of Woo et al. [15,50] showed that upon binding of a ligand to a membrane receptor an SRC family kinase is activated. This SRC kinase activates NADPH oxidase in the plasma membrane, which leads to the production of superoxide that dismutates into H_2_O_2_, and also catalyzes Prx1 phosphorylation at a tyrosine residue. This leads to inactivation of Prx1, due to a decreased reactivity of its catalytic cysteine residue with H_2_O_2_, and to a transient accumulation of H_2_O_2_ around membranes, where signaling components are concentrated. The increased levels of H_2_O_2_ promote further phosphorylation and inactivation of Prx1 both by activating SRC kinases and by inactivating PTPs. There is no cellular toxicity because this increase in H_2_O_2_ concentration occurs locally and any H_2_O_2_ that diffuses from this region will be degraded by active Prx1 and other peroxidases present in the cytoplasm. It should be mentioned that not all antioxidant systems may be present at the site of H_2_O_2_ production; for example glutathione peroxidase is not found in the sub-membrane fraction where H_2_O_2_ is produced [50]. The lag time in Prx1 catalysis caused by its phosphorylation is inversely proportional to the concentration of H_2_O_2_. This suggests that reactivation of Prx1 resumes when H_2_O_2_ levels rise beyond a certain threshold contributing to the termination of the signaling process [52]. Recently two other kinases, Mst1 and Mst2, which are both activated by H_2_O_2_, were shown to inhibit Prx1 [53]. Both these studies have an important general implication for H_2_O_2_-dependent redox regulation since they also suggest that phosphorylation/dephosphorylation of thiol proteins can alter their reactivity with H_2_O_2_ and so, we could speculate that an H_2_O_2_ sensor with low reactivity can become a high reactivity sensor and *vice-versa*, depending on its phosphorylation state.

A note should be made regarding the possible role GSH may have in mediating or modulating H_2_O_2_ signaling. GSH is several orders of magnitude more abundant than a low-reactive thiol protein such as PTP1B, and has a rate constant for the reaction with H_2_O_2_, 0.87 M^−1^ s^−1^ at pH 7.4, that is about 20–40 times lower than that of PTP1B. Taking into account these data, can GSH be considered a sensor molecule for H_2_O_2_ or can it inhibit H_2_O_2_ signaling mediated by PTP1B? The answer in both cases is no. In terms of reaction with H_2_O_2_, GSH certainly outcompetes PTP1B (Fig. 2), but the non-enzymatic reaction of H_2_O_2_ with GSH is negligible when compared with enzymatic systems removing H_2_O_2_, like catalase, GSH peroxidase or peroxiredoxins. Thus, the non-enzymatic reaction of GSH with H_2_O_2_ does not affect significantly the intensity of the H_2_O_2_ signal, and does not inhibit PTP1B-mediated H_2_O_2_ signaling. Concerning the non-enzymatic oxidation of GSH, this does not represent a signaling event because the product of this reaction does not relay information into a signaling pathway. It could be argued that an increased GSSG concentration would affect signaling by changing the ratio 2 × [GSH]/[GSSG], but the contribution of the non-enzymatic oxidation of GSH towards this ratio is negligible when compared with the enzymatic oxidation of GSH.

So far we have been discussing H_2_O_2_ signaling mediated by its direct reaction with thiol proteins, but alternative mechanisms have been described, involving H_2_O_2_-dependent formation of other second-messengers. For example, H_2_O_2_ formed in the mitochondria may initiate lipid peroxidation to produce reactive electrophilic lipid oxidation products that can act as second messengers leading to the activation of mitogen-activated protein kinases [54]. This initiation of lipid peroxidation may be mediated by heme proteins such as cytochrome *c* [55] and, in general, because of the high-reactivity of heme iron with H_2_O_2_, heme proteins could potentially act as H_2_O_2_ sensors. We could also speculate that the localized production of H_2_O_2_ in entrapped membrane compartments during signaling could initiate lipid peroxidation and form reactive lipid species, which has been suggested to have a signaling role [56].

From the data in Table 1 it becomes obvious that H_2_O_2_ signaling can operate either mediated by localized high transient levels of H_2_O_2_ that activate sensors with a low reactivity, or mediated by proteins with high reactivity towards H_2_O_2_ that work as the initial sensor that subsequently activate a low reactivity protein. Experimental support of both these mechanisms does exist [29,50] (Fig. 4).

## Biological functions of transcription factors regulated by H_2_O_2_

Before addressing the known mechanisms of H_2_O_2_-regulated TFs we briefly describe their biological functions. Particular attention is given to three evolutionary aspects that allowed H_2_O_2_ to evolve as a regulatory molecule. First, we address the constraints imposed by the lack of compartmentalization in bacteria, then the appearance of sub-cellular compartments in eukaryotes and, finally, the impact of multicellularity for H_2_O_2_ signaling. As a result, as we progress throughout the phylogenetic tree there is an increase in the complexity of the regulatory networks, involving TFs that are able to respond to variations of H_2_O_2_ levels.

### Bacterial transcription factors as direct sensors of H_2_O_2_

In organisms without cellular compartments, one way of achieving signaling is to use highly reactive proteins able to sense H_2_O_2_ and trigger a response. This is obligatory if a fast response is required. In fact, in the case of bacteria we highlight two TFs, OxyR and PerR, that are both directly regulated by H_2_O_2_. This duo of TFs, which are highly reactive with H_2_O_2_, display only one regulatory mechanism layer, which rapidly allows bacteria exposed to increasing levels of H_2_O_2_ to cope with oxidative damage increasing cell fitness and survival. The small size of bacteria, with the corresponding high ratio between surface area and volume make them particularly susceptible to environmental stresses, including H_2_O_2_. For example at anoxic/oxic interfaces oxidation reactions involving reduced metal ions and sulfur species that enter in contact with oxygenated waters produce H_2_O_2_. H_2_O_2_ is also formed when UV/visible radiation illuminates extracellular chromophores, including photosynthetic pigments that are released by decomposing plants. Also, H_2_O_2_ may be intentionally produced by competing organisms like lactic acid bacteria [57]. Thus, the capacity to rapidly respond to increasing concentrations of H_2_O_2_ will probably provide a survival advantage in various ecosystems.

#### OxyR

OxyR is a member of the LysR family of TFs that contains a conserved N-terminal helix-turn-helix DNA binding domain, a central co-inducer recognition and a response domain that senses the regulatory signal, and a C-terminal domain that is required for multimerization and activation [7,58,59]. In *E. coli*, tetrameric OxyR binds to the 5′ promoter–operator regions of target genes at a conserved sequence motif that contains four ATAG elements spaced at 10 bp intervals [60]. OxyR binds to DNA, either in its oxidized or in its reduced form, but only activates transcription when oxidized [61]. In the oxidized form OxyR contacts the DNA motif in four adjacent major grooves on one face of the DNA helix while the reduced form of OxyR binds DNA in two pairs of major grooves separated by one helical turn [60]. Most of the OxyR up-regulated genes are involved in defense systems against oxidative stress [7,58].

#### PerR

The Peroxide Regulon Repressor (PerR) is a metal-dependent TF and a major regulator of the peroxide inducible stress response in bacteria [[62], [63], [64]]. PerR was identified in 1998 and found to be a member of the ferric uptake repressor (Fur) family of proteins [62,65]. Unlike most members in the Fur family, PerR is not involved in metal homeostasis and, like OxyR, is a specific sensor of H_2_O_2_ [66]. In fact, PerR is a functional equivalent for OxyR and substitutes OxyR in many Gram-positive bacteria, although it may also coexist with OxyR [67]. However, like the other Fur family proteins, PerR DNA binding is also activated by a metal ion, either Fe^2+^ or Mn^2+^. PerR interacts with DNA at the *per* box, a specific palindromic consensus sequence (TTATAATNATTATAA) residing within and near the promoter sequences of PerR-controlled genes. In *Bacillus subtilis*, PerR, when bound to DNA, represses the genes coding for proteins involved in the oxidative stress response (*katA*, *ahpC,* and *mrgA*) [62,68], metal homeostasis (*hemAXCDBL*, *fur*, and *zosA*) [[68], [69], [70]] and its own synthesis (*perR*) [69]. Most PerR-regulated genes are de-repressed in cells treated with low levels of extracellular H_2_O_2_ (8 µM) [64] or cells cultured under conditions of iron and manganese ions deficiency [69].

### The challenge of cellular compartmentalization in lower eukaryotes

In yeast, a eukaryote but still a unicellular organism, we will focus on the analysis of four TFs, namely, Yap1, Maf1, Hsf1, and Msn2/4. In this group Yap1 is regulated by H_2_O_2_ at the level of cytoplasm/nucleus traffic, which creates a new layer on the regulatory mechanisms when compared with OxyR and PerR. Thus, though Yap1, like OxyR and PerR, essentially allows cells to deal with oxidative stress response, the complexity of its regulatory mechanism already reflects cell compartmentalization. This regulatory layer is also found in Maf1, but the traffic regulator partners are now replaced by post-translational modifications (PTMs) that create different intracellular pools of the protein and determine its subcellular localization. In the case of Hsf1 and Msn2/4, both TFs allow cells to respond to a variety of different environmental stresses from heat shock to starvation and oxidative stress. Thus, in yeast, the response to oxidative stress is also part of a more general cellular response to stress, probably making this response to oxidative damage more robust.

Compartmentalization creates new opportunities to generate new levels of regulation and confine certain pathways and metabolic pathways to specific compartments. Moreover, yeast cells are larger than bacteria and this may have contributed to create endogenous H_2_O_2_ gradients between distinct compartments [44]. In the case of cytoplasm/nucleus the appearance of these two compartments allowed DNA to be less susceptible to oxidative damage contributing to distinguish H_2_O_2_ toxic and regulatory responses, thus facilitating evolution of H_2_O_2_ as a regulatory molecule. Also, endogenous sources of H_2_O_2_, such as dismutation of superoxide produced by NADPH oxidase (NOX) enzymes began to be established. Up until recently, it was believed that the genomes of *Saccharomyces cerevisiae* (*S. cerevisiae*) and *Schizosaccharomyces pombe* (*S. pombe*) did not contain genes encoding NOX enzymes. Recently, Rinnerthaler et al. [71] showed that one of the *S. cerevisiae* ORF encodes an authentic NOX, which is located in the endoplasmic reticulum (ER) membrane and produces superoxide in a NADPH-dependent fashion. Interestingly, most NOX enzymes are plasma membrane bound but, NOX4 has been localized in endomembranes of ER, the nucleus and, in spite of some contradictory data, in the mitochondria [72]. This clearly shows that the appearance of the intracellular membranes associated to new compartments contributed to the evolution of a regulatory role for H_2_O_2_.

#### Yap1

Yap1 (yeast AP-1) is one of the members of the yeast *S. cerevisiae* activator protein (Yap) family that comprises eight members [73]. All members display a significant sequence similarity at the DNA-binding domain, the basic leucine zipper (b-ZIP domain) in the N-terminus [74]. Structurally the Yap1 factor has two cysteine residues rich domains (CRD), the nCRD (Cys303, Cys310 and Cys315) and cCRD (Cys598, Cys620 and Cys629) located in the N- and C-terminal, respectively [75]. Under oxidative stress, nuclear export of Yap1 is decreased and Yap1 is retained in the nucleus where it can regulate its target genes [76]. Yap1 has a key role in the oxidative stress response, redox homeostasis and electrophilic response, regulating the transcription of genes encoding antioxidant and detoxification enzymes.

#### Maf1

Maf1 is a transcriptional repressor of RNA polymerase III (Pol III) that was originally discovered in *S. cerevisiae* [77]. However, Maf1 is also found in human, animals and plants [78]. In yeast, Pol III is responsible for the transcription of around 300 different genes, mostly tRNA genes [79]. Maf1 does not bind directly to DNA; instead it binds to Pol III clamp and rearranges the subcomplex C82/34/31, which is required for transcription initiation [80]. In this repressive complex, Maf1 impairs recruitment of Pol III to a complex of promoter DNA with the initiation factors TFIIIB and thus prevents formation of a closed-complex.

Maf1 is a hydrophilic protein conserved from yeast to human and it contains three signature domains not found in any other polypeptide: A, B and C boxes [81]. Yeast Maf1, contrary to human MAF1, contains two conserved nuclear localization signals (NLS) [77]. Maf1 activity is regulated by means of its phosphorylation state-dependent cellular localization [78].

#### Hsf1

In eukaryotic cells, the heat shock response is primarily mediated by a homotrimeric DNA-binding TF, the heat shock factor 1 (HSF1), a member of the heat shock TF family that binds to *cis*-acting promoter elements in target genes, called heat shock elements (HSE) [82]. Each HSE contains two, or more, contiguous inverted repeats of the 5-bp sequence nGAAn. HSF1 is regulated differently in mammalian cells and in yeast [83].

The *S. cerevisiae* HSF1 homologue, Hsf1, is constitutively a trimer, and is localized in the nucleus where it associates with high-affinity HSEs under normal conditions and additional HSEs during stress [83,84]. The structure of Hsf1 is highly conserved with its mammalian homologue [82]. Besides being involved in the heat shock response several lines of evidence have shown that Hsf1 is also involved in the yeast oxidative stress response [85]. Target genes of Hsf1 encode for molecular chaperones such as heat shock proteins (HSPs), metabolic enzymes, and cell wall proteins [86].

#### Msn2/4

Msn2 and Msn4 (Msn2/4) are homologous and functionally redundant Cys2His_2_ zinc finger yeast TFs [87]. In *S. cerevisiae*, disruption of both *MSN2* and *MSN4* genes results in a higher sensitivity to different environmental stresses, including carbon source starvation, heat shock and severe osmotic and oxidative stresses. Msn2/4 are required for activation of several yeast genes, whose induction is mediated through the presence of a stress responsive element (STRE) consisting of a pentameric core of CCCCT, such as *CTT1*, coding for cytosolic catalase, and *HSP12* [87].

### The challenge of multicellularity in higher eukaryotes

The complexity of the next step in eukaryotes evolution is attained by acquisition of multicellularity and, therefore, the appearance of different cell types and tissues. Cells are now able to differentiate and gain very specialized functions, their proliferative organization is diverse and cells must integrate cell–cell and cell–matrix connections. Some cells maintain their ability to be totipotents, and multicellularity is now upstream of cell compartmentalization. Cells are now exposed to a new environment and information is received from this environment mainly *via* receptors in the plasma membrane.

Interestingly, in this new scenario, H_2_O_2_, despite still having the capacity to cause damage, acquires a prominent role as a regulatory molecule. Now, cells do not only respond to environmental H_2_O_2_, but H_2_O_2_ evolved as a second messenger necessary for many signaling pathways. Therefore, although the stress-response strategies found in yeast for the regulation of transcriptional factors by H_2_O_2_ can be detected in multicellular organisms (for example, HSF1 and NRF2), now evolution expanded this to an incredible complexity and invented new regulatory mechanisms and combinations between the pre-existent mechanisms allowing each protein to have different code bars and, therefore, integrate different signaling pathways and compartments. In a few cases, the TF is even part of a membrane receptor and is released upon activation, playing membranes a critical role.

In multicellular organisms we focused our attention in nine different transcriptional factors, namely AP-1, NRF2, CREB, HSF1, HIF-1, TP53, NF-κB, NOTCH, SP1 and SCREB-1. It should be noted that some of these TFs (*e.g.* TP53, NF-κB, CREB and SCREB) that are relevant to metazoan multicellularity evolved prior to the emergence of the metazoan stem lineage, and they can be found in some transition organisms, such as choanoflagellates or *Capsaspora owczarzaki*, but not in other lower unicellular eukaryotes such as yeast [88]. Noteworthy, most of them are involved in the regulation of cell damage response, cell proliferation (cell cycle regulation), differentiation and apoptosis (AP-1, CREB, TP53, NOTCH, NF-κB, and SP1). Therefore, they are closely linked to cell survival and development, and their deregulation is in the basis of different pathophysiological stages such as cancer. HIF-1 and SREBP-1 seem to have a narrow range of actions essentially controlling lipid metabolism and O_2_ levels at the cellular and systemic level. Like it happens in yeast, HSF1 still orchestrates the cellular response to a variety of cellular stresses, and H_2_O_2_ is still under the surveillance of general cell protecting mechanisms. The ability of certain TFs for transactivating response genes still recalls OxyR since they directly sense H_2_O_2_ as is the case of human HSF1.

The way H_2_O_2_ regulates the activity of these transcriptional factors is diverse but clearly explores the existence of different cellular compartments, and enrichment in different biochemical forms of both the TF protein and its partners, by PTMs and different forms of processing. In all these different levels of regulation we find targets for the regulatory action of H_2_O_2_.

#### AP-1

Activator protein-1 (AP-1) is a TF that regulates several cellular processes, including cell proliferation, apoptosis, survival, and differentiation. Such functional diversity derives primarily from its structural and regulatory complexity [89]. The term AP-1 describes a collection of dimeric bZIP proteins, mainly from the Jun (v-Jun, c-JUN, JUND, and JUNB), Fos (v-Fos, c-FOS, FRA-1, FRA-2, and FOSB), ATF/CREB (CREB, ATF1, ATF2, ATF4, ATF5, ATF6a, ATF6b, ATF7, ATF3/LRF1, B-ATF, and ATFa0), JDP (JDP1/2), small MAF (MAFG, MAFF, and MAFK) and large MAF (cMAF, MAFB, MAFA, and NRL) sub-families that usually form heterodimers that bind to a TPA-responsive element (TRE, 5′ TGAG/CTCA-3′) or cAMP response elements (CRE, 5′-TGACGTCA-3′) [90,91]. In these TFs the basic region of the bZIP domain mediates DNA binding, whereas the leucine zipper is responsible for dimerization with the partner bZIP factor.

#### NRF2

The NRF protein family constituted by NRF1, NRF2, and NRF3, which are also bZIP proteins, regulate electrophilic xenobiotic detoxification and oxidative stress response. The main activators of this TF are electrophile agents but H_2_O_2_ also activates NRF2 [92] by a multitude of mechanisms. NRF proteins bind to the electrophile response element (EpRE, 50-(A/G)TGACNNNGC(A/G)-30) in the target genes, cannot form homodimers, and the typical partners are small MAF proteins, although c-JUN has been reported to heterodimerize with NRF2 [90,93]. In this review, we will focus on NRF2.

#### CREB

The cAMP response element-binding protein (CREB) is one of the three members of the cAMP responsive TFs family occurring in mammals (CREB, CREM and ATF-1). These TF play important roles in the nuclear responses to a variety of external signals, by binding different promoters of genes encoding proteins involved in transcription, metabolism, cell proliferation, differentiation, apoptosis, and the secretory pathway [94]. CREB binds as a homodimer to the CRE conserved TGACGTCA sequence [95]. This 43 kDa TF has a dimerization and DNA binding, and an N-terminal activation domain (AD) with two independent regions: the phosphorylation box (P box) and a second region comprising two glutamine-rich domains, Q1 and Q2, which flank the P box [96]. The P box contains a cluster of sites phosphorylated by various kinases that regulate the transactivation potential of this protein [95]. Phosphorylation of CREB, mainly at Ser133, enables the recruitment of the co-activators CBP/p300 and stimulates CREB-dependent transcription [95]. However, CREB activity depends on other regulatory partners that are required for recruitment of the transcriptional apparatus to the promoter. More than 20 different protein kinases, members of distinct signaling pathways, have been described as CREB kinases [95,97]. The activity of CREB as a TF can be regulated by other PTMs such as acetylation, ubiquitination, sumoylation and glycosylation [95].

#### TP53

TP53 (Tumor protein TP53, TTP53, Li–Fraumeni syndrome 1) has been studied for nearly three decades, and is best known for its potent ability to be a tumor suppressor [98]. In fact, this protein is encoded by the *TTP53* gene that commonly has lost its function by mutations in the majority (75% of the cases) of human cancers.

TP53 is a DNA-binding TF that both activates and represses a broad range of target genes constituting a critical hub that integrates a huge variety of signals and allows a complex set of cellular responses to DNA damaging agents, oxidative stress, oncogene activation (deregulated growth signals), mitotic spindle damage, hypoxia, nutrient deprivation, telomere erosion, ribosomal stress and is involved in cellular senescence [[99], [100], [101], [102]]. The TP53 protein comprises different structural and functional domains. The N-terminal domain corresponds to the transactivation domain required for transcriptional regulation of the target genes. This domain contains a proline-rich region that plays a role in the regulation of TP53 stability by the negative regulator protein murine double minute 2 (MDM2) [103]. The central domain binds in a zinc-dependent manner [104,105] to a consensus site that shows an internal symmetry and is composed by two copies of the sequence 5′-PuPuPuC(A/T) (T/A)GPyPyPy-3′ separated by 0–13 bp [106,107]. The C-terminal region of the protein contains the oligomerization domain (tetramerization) where specific signals for nucleus import/export are localized [108]. This domain catalyzes DNA annealing and strand transfer and displays a strong preference for damaged DNA by ionizing radiation, having thus specialized functions [109].

#### NOTCH

The receptors and ligands in the NOTCH signaling pathway are membrane proteins that imply cell–cell contact for their activation, and they constitute the basis of NOTCH-dependent transcription activation. Mammals express four receptors, NOTCH1, NOTCH2, NOTCH3 and NOTCH4 and two families of ligands, Jagged (Jagged1 and Jagged2) and Delta-like (Dll1, Dll3 and Dll4) [110]. The canonical model for NOTCH signaling activation requires a crucial proteolysis that releases the NOTCH intracellular domain (NICD) from the plasma membrane after ligand activation expressed in a neighboring cell. NICD cleavage is mediated by the γ-secretase complex and is facilitated by the previous proteolytic cleavage of NOTCH extracellular domain (NECD) by a metalloproteinase ADAM17/TACE [111]. NICD is then translocated to the nucleus where it associates with the DNA-binding protein Suppressor of Hairless (SU(H)) and with the nuclear effector Mastermind (MAM) for transcriptional activation [110]. The NOTCH signaling pathway has been implicated in numerous cellular processes including neuron differentiation and blood vessel formation in normal embryo development and in disease [112].

#### NF-κB

The NF-κB/REL family of TFs has key regulatory roles in inflammation, innate and adaptive immune response, proliferation and apoptosis [113]. It consists of homo- and heterodimers of five distinct proteins, the REL subfamily proteins (p65/RELA, RELB, and c-REL), which contain C-terminal transactivation domains (TADs) and the NF-κB subfamily proteins (p50, and p52, and its precursors p105 and p100, respectively) [113]. All NF-κB/REL proteins contain a Ref-1-homology domain (RHD) that also harbors an NLS, which is responsible for dimerization, recognition and binding to DNA and also for the interaction with the inhibitory proteins IκBs [114]. The IκB family is composed of IκB-α, IκB-β, IκB-ϵ, IκB-γ and BCL-3 (B-cell lymphoma 3) possessing typical ankyrin repeats that mediate binding to the RHD and interfere with its NLS function. The most common composition of cytoplasmic NF-κB/IκB complex appears to be the p50/p65/IκB-α [114].

The IκB proteins bind to NF-κB in the cytoplasm preventing NF-κB translocation to the nucleus and its binding to DNA. Therefore, complexation with IκBs has to be removed for NF-κB activation [115]. NF-κB activators such as tumor necrosis factor α (TNF-α), lipopolysaccharide (LPS) and interleukin-1 (IL-1) activate the IκB-kinase complex (IKK complex), which catalyzes the phosphorylation of IκBs at specific regulatory amino acid residues. As a consequence, the IκBs are targeted for degradation by the 26S proteasome thereby freeing NF-κB, which translocates to the nucleus and binds to the promoter/enhancer regions of target genes, the κB sites, which have the general consensus sequence GGGRNNYYCC (R is a purine, Y is a pyrimidine, and N is any base) [113]. Target genes include pro-inflammatory cytokines, chemokines, adhesion molecules, growth factors, and enzymes that produce secondary inflammatory regulators such as cyclooxygenase-2, inducible NO synthase, and heme oxygenase [22,116].

#### SP1

SP1 (Specificity protein 1) was the first TF to be identified, purified and cloned from mammalian cells [117]. SP1 is a member of an extended family of DNA-binding proteins that have three zinc-fingers (Cys2His_2_ – type zinc finger), which are required for recognizing GC-rich promoter sequences [118,119]. SP1 contains two glutamine-rich domains that are essential for transcriptional activation. Next to these domains are serine/threonine-rich sequences that may be a target for PTMs [119]. SP1 is an essential TF that can activate or repress transcription in response to physiological and pathological stimuli, such as oxidative stress [120,121]. SP1, besides regulating itself, is also implicated in the regulation of many genes that play important roles in a variety of physiological processes including cell cycle regulation and growth control, hormonal activation, apoptosis, and angiogenesis [122].

SP1 directly interacts with TATA-binding protein associated factors [123] and other factors, such as those binding to cAMP response elements [124], NF-κB [125] and vascular endothelial growth factor receptor-2 (VEGFR-2) [126]. The activity of SP1 as a TF can be regulated by PTMs such as phosphorylation, acetylation and methylation [120,127] that regulate SP1 protein level, transactivation activity, and DNA binding affinity [128].

#### HIF-1

HIF-1 (hypoxia-inducible factor) is a TF that has an essential role in the response to hypoxia at systemic and cellular level. This TF has been implicated in the activation of angiogenesis and erythropoiesis [129,130] and in the metabolic adaptation to hypoxia through activation of glycolysis [131]. HIF-1 is a dimeric protein complex formed by an inducible subunit (HIF-1α) and a constitutive subunit (HIF-1β) that are basic helix-loop-helix (bHLH) proteins. The HIF-1α/β dimer binds to a DNA motif (G/ACGTG) in hypoxia-response elements (HREs) of target genes [132].

HIF-1α contains two hypoxia-dependent degradation domains with two conserved prolyl residues. The hydroxylation of these residues catalyzed by PHD promotes the interaction between HIF-1α and the Hippel–Lindau tumor suppressor (pVHL), targeting the former for proteasomal degradation [28,133] . Although the activation of HIF-1 has been mainly related with low levels of O_2_, this TF can also be activated by a hypoxia-independent mechanism that is mediated by the superoxide radical and by H_2_O_2_ [134,135].

#### SREBP-1

Sterol regulatory element binding proteins (SREBPs) are a family of critical TFs that bind the sterol regulatory element (SRE), activating genes encoding the enzymes that regulate the synthesis of cholesterol, lipids and fatty acids and cellular uptake of lipoproteins [[136], [137], [138]]. H_2_O_2_ has a strong influence on SREBP1 activity in cells with a high sensitivity to insulin, promoting lipid accumulation [139]. There are three SREBP isoforms, designated SREBP-1a, SREBP-1c, and SREBP-2 [138]. SREBP proteins, initially synthesized as a 125 kDa membrane bound precursor, are anchored to the ER [136,140]. They share a similar tripartite structure: an N-terminal region, which has a TF domain of the basic helix-loop-helix-leucine zipper (bHLHZip) family, a central domain, which contains the two transmembrane spans, and a C-terminal regulatory domain that binds tightly to the C-terminal domain of SREBP cleavage activating protein (SCAP) [141,142]. The complex SREBP–SCAP is maintained in the ER *via* the interaction with the protein INSIG (Insulin-induced gene), which binds directly the protein SCAP [143]. Despite differences in their transcriptional targets, the proteolytic activation of each SREBP isoform is regulated by cholesterol and oxysterols through a common mechanism [144,145]. In the presence of these compounds, SREBP–SCAP is retained in the ER by binding to Insig, contrary to what happens in the absence of sterols [143] where Insig no longer binds SREBP–SCAP and SREBP–SCAP is translocated to the Golgi [143]. Once in the Golgi, the SREBP active form is obtained after two sequential proteolytic cleavages of the SREBP precursor form, mediated by distinct site specific proteases, namely Site-1 protease (S1P) and Site-2 protease (S2P) [146], in order to release the amino-terminal TF domain of SREBP from the membrane [142]. The activated N-terminal domain of SREBP translocates into the nucleus to bind the SRE (ATCACCCCAC) sequence and the E-box (CAXXTG) sequence of the promoter of target genes and trigger gene expression [139].

#### HSF1

The mammalian heat shock factor (HSF) family has four members HSF, HSF2, HSF3 and HSF4 but HSF1 is the key stress-responsive regulator of the heat shock response [82]. HSF1 has several functional domains, the N-terminal DNA-binding domain, the oligomerization domain containing heptad repeat regions HR-A and HR-B that regulates trimerization, the regulatory domain and the C-terminal trans-activation domain [82,147]. An additional heptad domain region, HR-C, that maintains HSF1 in an inactive state by suppressing spontaneous trimerization is located between the regulatory and trans-activation domains. The DNA-binding domain is of a looped helix-turn-helix type but, unlike TFs containing similar domains, HSF1 does not make direct contact with DNA [147]. The loop apparently stabilizes the DNA-bound HSF1 trimer by protein–protein interactions. The regulatory domain in HSF1 negatively regulates the transactivation domain and is targeted by several PTMs including phosphorylation, sumoylation and acetylation [147].

HSF1 is constitutively expressed in most tissues and cell lines [82]. In the absence of stress conditions HSF1 exists as a monomeric phosphorylated protein that interacts with HSP90 and is present in both the nucleus and cytoplasm [82]. In mammalian cells, on exposure to diverse stress conditions, including oxidative stress, monomeric HSF1 undergoes a multistep activation process that includes dissociation from the Hsp90 complex, trimerization, nuclear accumulation, PTMs, DNA binding and target gene activation [82,147]. The activation of HSF1 is also regulated by binding to heat shock proteins at different phases of the activation process [82]. For example*,* elevated levels of both Hsp70 and Hsp90 regulate HSF1 through a negative feedback preventing trimer formation during heat shock [148]. Also, Hsp70 and Hsp40 interact with activated HSF1 trimers to inhibit transactivation [[148], [149], [150]].

## Redox regulation of transcription factors by H_2_O_2_

The activity of TF can be regulated by two mechanisms: (i) by synthesis/degradation, where upon activation they are synthesized *de novo* or their stability increases, and (ii) by controlling the activity of a pre-existent TF. There are different mechanisms by which TFs can be activated from an inactive to an active form, most of them meditated by PTMs: state of oligomerization, by binding to a ligand, dissociation from an inhibitory protein, cleavage of a larger precursor, cellular relocation, and access to promoter regions. In principle, activation of a pre-existent TF allows for a faster response to stimuli in order to alter the activity of cellular TFs and produce alterations in gene expression, but, for example, mobilization of a pre-existent mRNA for *de novo* synthesis is also a rapid process [151]. In what follows we analyze how H_2_O_2_ regulates TFs at each of these levels – synthesis of TF, degradation of TF, cytoplasm-nuclear trafficking, and DNA binding and transactivation – illustrating with representative examples of known regulatory mechanisms.

### Protein synthesis of the transcription factor

**Fig 5:**
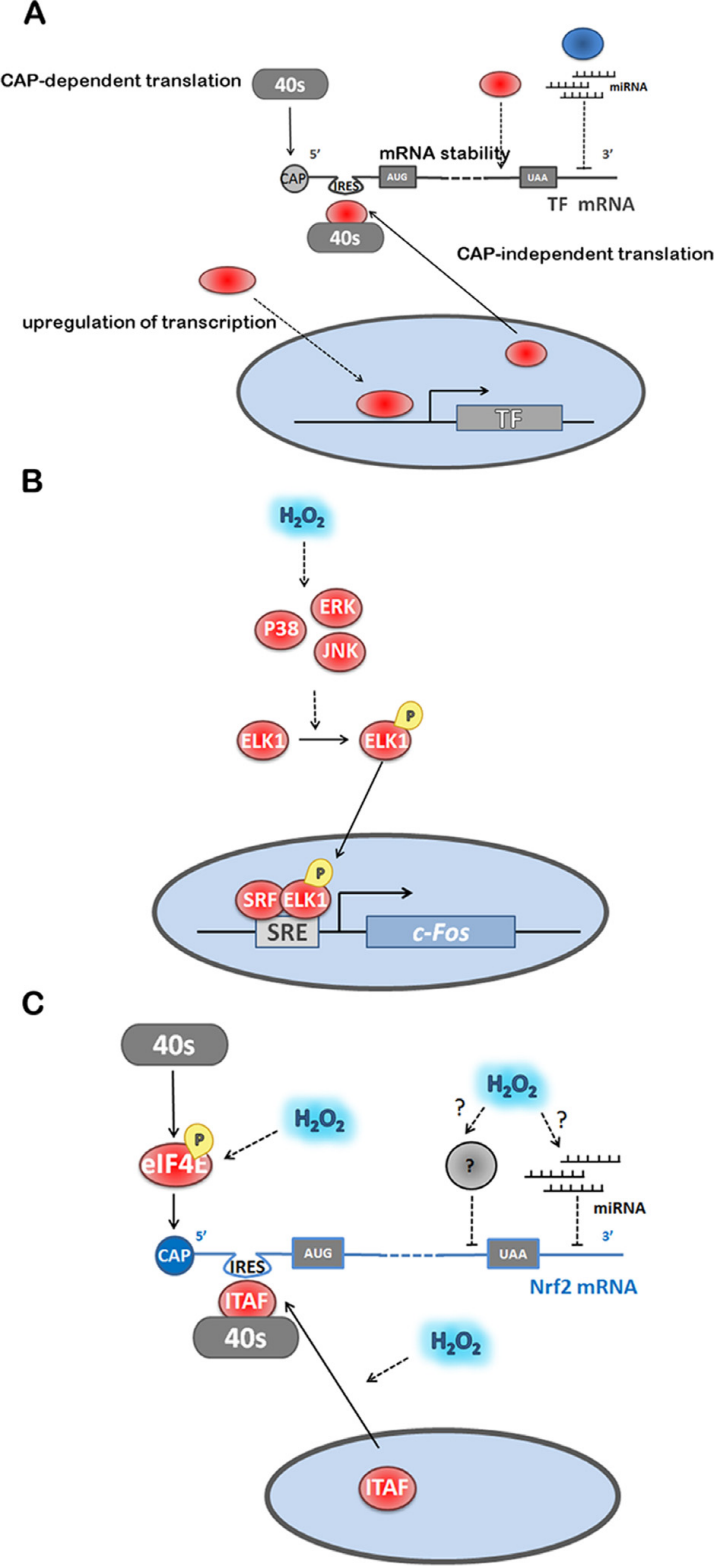
Regulation of TF expression by H_2_O_2_. **(A)** TF expression is regulated by H_2_O_2_ at both transcriptional and translational levels. The translation process and the regulation of mRNA stability are preferential targets of H_2_O_2_. H_2_O_2_ modulates CAP-dependent and independent translation through the activation of 40S-mRNA complexes. mRNA stability is modulated by RNA-binding proteins and by specific miRNA, which are modulated by H_2_O_2_. **(B)** c-FOS transcription is regulated by ELK1, which is phosphorylated by MAP kinases activated by H_2_O_2_. **(C)** The known mechanism for NRF2 is exemplified showing the positive regulation by H_2_O_2_ of both CAP-dependent and CAP-independent initiation. The ITAF for NRF2 is La Autoantigen, whose translocation to the cytoplasm is promoted by H_2_O_2_. Also shown, are hypothetical mechanisms for H_2_O_2_ modulation of microRNAs that negatively control translation by binding to the 3′ UTR region of NRF2 mRNA, and of unknown factors that mediate repressing of NRF2 translation by binding to the 3′ part of the mRNA coding regions. Factors colored blue are inhibitors of TF-dependent gene expression; factors colored red are activators of TF-dependent gene expression. Dashed lines indicate activation/inhibition.

H_2_O_2_ regulates several TFs by upregulating their synthesis at the transcriptional, post-transcriptional and translational levels (Fig. 5A). In fact, H_2_O_2_ increases the rate of transcription of AP-1, TP53 and HIF-1α, increases the rate of translation of Nrf2 and SP1 or may also regulate TP53 mRNA stability.

#### Upregulation of transcription

AP-1, when upregulated, spontaneously concentrates in the nucleus to activate gene expression [152,153]. Upregulation by H_2_O_2_ of both c-JUN and c-FOS is done at the transcriptional level by activating the mitogen-activated protein kinase (MAPK) subgroups c-JUN amino-terminal kinase (JNK), p38 MAPK and extracellular signal-regulated protein kinase (ERK) [154]. c-JUN is one of the target genes of Jun/AP-1 and so upon transactivation induced by JNK (see Transactivation and Binding section), c-JUN levels increase in a positively autoregulated loop [155]. JNK, p38 MAPK and ERK are all responsible for the transcriptional activation of c-FOS, because these kinases phosphorylate and activate ELK-1, resulting in enhanced serum-response element-dependent c-FOS expression [156] (Fig. 5B).

Several studies have suggested that TP53 protein levels increase in response to a rise of intracellular H_2_O_2_ [[157], [158], [159], [160]]. This may occur through regulation of TP53 transcription mediated by other H_2_O_2_-dependent TFs. The *TP53* gene is positively and negatively regulated at the transcriptional level from several promoters having different strengths [161]. One such promoter, designated TP53P1, contains several responsive elements for the H_2_O_2_-regulated TFs AP-1 and NF-κB, which bind c-JUN/c-FOS and p50-p65 (NF-κB1–RelA) respectively, and are required for efficient transcription of TP53 in human cells [162]. TNF-α-induced activation of NF-κB also activates TP53 by specific recognition of the NF-κB site in the TP53 promoter [163]. Since H_2_O_2_ synergistically increases TNF-α-induced activation of NF-κB [42,116,164] it should be expected that H_2_O_2_ would also affect NF-κB-dependent TP53 transcription activation.

Another TF that is activated at the transcriptional level is HIF-1α. Activation of HIF-1α transcription by Angiotensin II, in vascular smooth muscle cells, involves the H_2_O_2_-dependent activation of the phosphatidylinositol 3-kinase (PI3K) pathway [165,166]. The H_2_O_2_ role in the induction of HIF-1α transcription might also be mediated by NF-κB in vascular smooth muscle cells [166]. NF-κB activates directly HIF-1α transcription upon recognition of an NF-κB binding site (at −197/−188 bp upstream of the transcription initiation site) in the HIF-1α promoter. Both an extracellular bolus addition of H_2_O_2_ (10–100 µM) and NOX4 overexpression increase HIF-1α mRNA levels [166]. Therefore, H_2_O_2_ acts as a general second messenger for HIF-1-dependent gene expression under normoxia conditions.

#### mRNA stability

There are several studies indicating that TP53 mRNA stability increases in response to cell stress conditions. The 3′- UTR of TP53 mRNA is a target for the RNA-binding protein HUR, which binds and stabilizes TP53 mRNA in response to short-wavelength UV light (UVC) [102]. Exposure of cells to H_2_O_2_ leads to an increase in cytoplasmic HUR levels [167] and HUR translocates from the nucleus to the cytoplasm and increases IL-6 and IL-8 mRNAs stability [168]. Consequently, it is plausible that H_2_O_2_ may also regulate TP53 mRNA stability through HUR.

#### Upregulation of translation

In general, upon exposure to a stress the overall protein synthesis is inhibited, while specific synthesis of stress response factors is upregulated. Protein translation is initiated in eukaryotic cells by two mechanisms: (i) CAP-dependent ribosome scanning, in which the eukaryotic initiation factor 4F complex (eIF4F), recruits the 40S ribosome that scans the 5′-untranslated region (UTR) until it finds the initiation codon AUG; and, (ii) CAP-independent internal ribosome entry mediated by internal ribosomal entry sites (IRESs). Normal physiological protein synthesis is mostly done *via* CAP-dependent mechanisms while protein synthesis of stress response factors is done *via* IRESs, which can induce cells to rapidly produce sufficient amounts of protein in response to the stress [169]. H_2_O_2_ upregulates both CAP-dependent and CAP-independent translation of TFs (Fig. 5A).

In addition to the transcriptional activation discussed previously, HIF-1α expression is also regulated at the translational level. H_2_O_2_ promotes the activation of a specific kinase for the S6 ribosomal protein, which is a component of 40S ribosomal subunit, p70S6k, increasing HIF-1α translational rate. This mechanism is induced by insulin and mediated by H_2_O_2_-dependent activation of MEK/ERK signaling [170].

Concerning NRF2, upregulation of its translation is an important regulatory control exerted by H_2_O_2_ [171], as a near 50% of maximal response is achieved after 5 min when HeLa cells are exposed to an H_2_O_2_ steady-state of 12.5 μM [172]. After applying Eq. (7), this fast response translates into a rate constant of 1.8 × 10^2^ M^−1^ s^−1^, for the reaction between H_2_O_2_ and the target(s) mediating this response. However, if a gradient of 6.8 between extracellular and intracellular H_2_O_2_ concentrations is considered [42], a rate constant of 1.3 × 10^3^ M^−1 ^s^−1^ is obtained instead. As this estimate is based on NRF2 protein levels, the sensor that triggers this pathway should actually have a higher rate constant, and thus we may speculate that it is a highly reactive H_2_O_2_ sensor.

The mechanism by which H_2_O_2_ stimulates NRF2 translation is both cap-dependent and independent [173] (Fig. 5C). CAP-dependent translation may be related to the stimulation of eIF4E phosphorylation at Ser209 by H_2_O_2_ [173]. The CAP-independent upregulation of NRF2 translation is mediated by an IRES sequence identified within the 5′ untranslated region of human NRF2 mRNA containing a highly conserved 18S rRNA binding site (RBS) complementary to the 749–761 bp of human 18S rRNA [173]. In general, IRES activity is regulated by IRES trans-acting factors (ITAFs), localized in the nucleus in the resting state, and which, upon a signal mechanism, translocate to the cytoplasm where they interact with IRES to recruit eIFs and ribosomes to initiate translation [174]. One of such ITAFs, La Autoantigen, was identified as being activated by H_2_O_2_ in HeLa cells; a treatment with 100 μM H_2_O_2_ for 10 min was sufficient to trigger nuclear export of this ITAF, followed by binding to the NRF2 5′ UTR and subsequent translation [175]. The mechanism by which H_2_O_2_ stimulates translocation of La Autoantigen to the cytoplasm is still unknown. Either dephosphorylation of Ser366 [176] or phosphorylation of Thr301 by AKT in mouse glial progenitor cells was reported to promote its cytoplasmic translocation [177], but it was also observed that (de)phosphorylation does not affect the subcellular localization of La Autoantigen [178]. In HeLa cells the phosphorylation status of the La Autoantigen was not observed to be under the influence of H_2_O_2_ [175].

Another example of H_2_O_2_-dependent regulation of the translation rate of a TF is SP1 in neurons. H_2_O_2_ formed extracellularly from d-amino acid oxidase, significantly upregulates both the protein levels and the DNA binding ability of SP1, and of its homologue Sp3, in cortical neurons *in vitro* and *in vivo* [120]. SP1 levels increase significantly 1-, 1.5- and 2-h after the addition of H_2_O_2_ to neuronal cultures, and H_2_O_2_ activates the IRES motif present in the 5′-UTR of SP1 mRNA, increasing SP1 levels through enhanced translation of the existing SP1 mRNAs, protecting neurons against ischemic damage [121]. Since SP1 levels auto-regulate its own transcription rate, the high levels of SP1 will lead to a later on increase of SP1 transcriptional rate. It is interesting to note that H_2_O_2_ activates SP1 translation only in neurons and not in glia cells and it has been proposed that neurons and glial cells probably have different ITAFs that respond differentially to H_2_O_2_.

The control of translation is a fast-moving area of research and in recent years a lot of information has been obtained. For example, the synthesis of AP-1 is regulated at the translational level, both by cap-dependent and independent mechanisms and by microRNAs [179]. Besides the IRES site present in the 5′-UTR region of NRF2, other regulatory elements are present in NRF2 mRNA. The 3′-UTR region is recognized by microRNAs that repress NRF2 translation [180], while the coding region of NRF2, more specifically in the segment 1159–1815 bp within the 3′ portion of the ORF, contains elements that repress NRF2 translation and are responsible for the low-basal levels of NRF2 synthesis (Fig. 5C) [181]. Finally, IRES sequences have been identified in TP53 [182], YAP1 [183] and c-JUN [179]. Modulation of these modes of control by H_2_O_2_ is still unknown, but new developments in this area are to be expected in the near future.

### Degradation of the transcription factor

Degradation of key modulators of TF function is an important regulatory mechanism in many signaling pathways. The majority of intracellular proteins are degraded *via* the ubiquitin (Ub)-proteasome pathway, which consists in the degradation of poly-ubiquitinated proteins by a multicatalytic protease called proteasome. Ub-protein ligase (E3) enzymes transfer the activated Ub from an Ub-conjugating protein E2, first to a lysine residue of the target protein and then to lysine residues present in the last added ubiquitin, yielding an Ub chain [184].

**Fig. 6:**
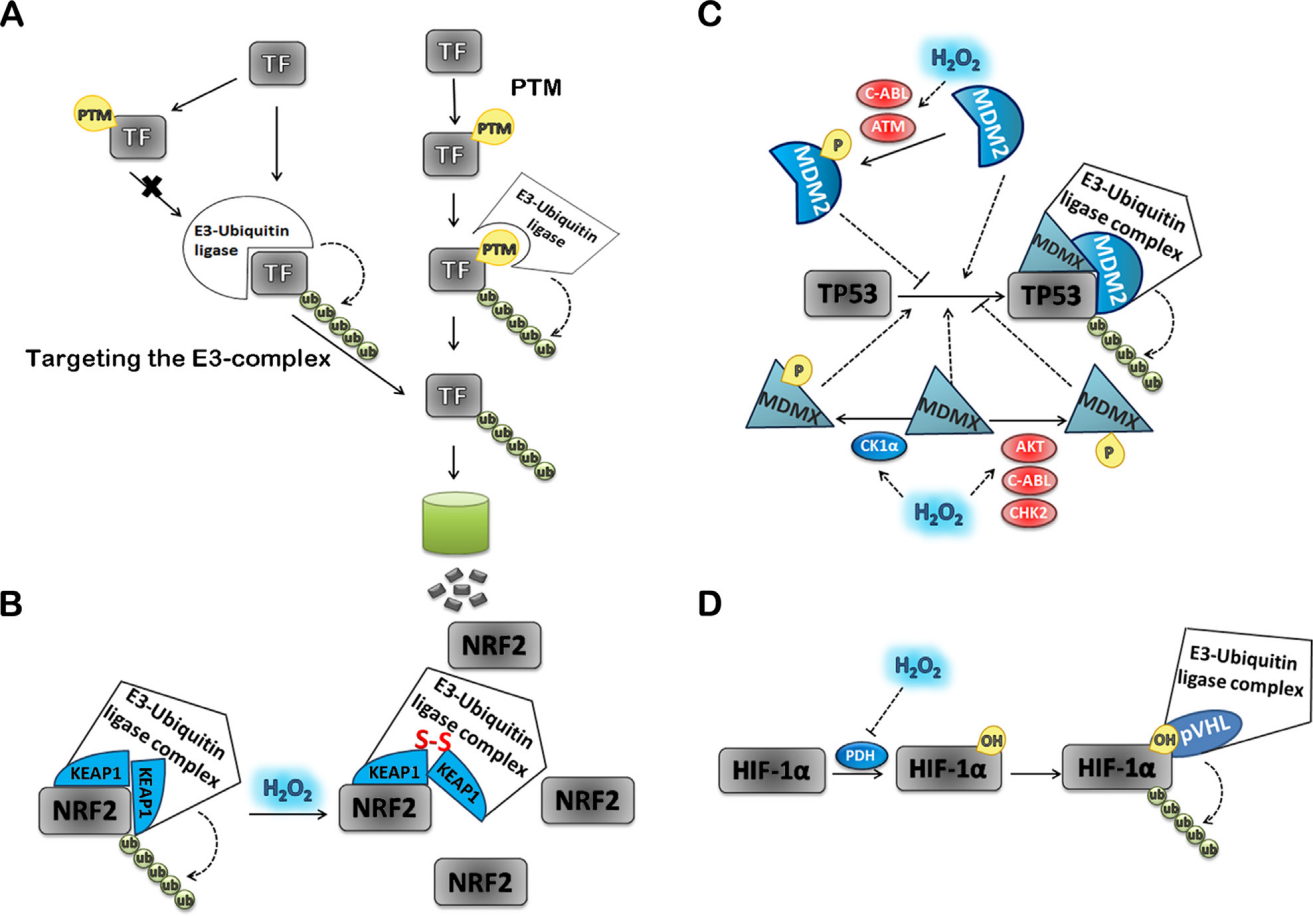
Regulation of TFs activity by degradation modulated by H_2_O_2_. TF degradation *via* poliubiquitin(Ub)-proteasome pathway is an important regulatory mechanism in different signaling pathways **(A)**. Proteasome, a multicatalytic protease oligomeric complex, degrades proteins that have been poly-ubiquitinated. Ubiquitin (Ub)-protein ligase (E3) enzymes transfer the activated Ub from an Ub-conjugating protein E2, first to a lysine residue of the substrate protein and the next Ub to lysine residues present in the last added ubiquitin, originating an Ub chain. NRF2 ubiquitination and degradation involves KEAP1 as the substrate adaptor subunit in the E3 holoenzyme **(B)**. In the presence of H_2_O_2_ critical cysteine residues in KEAP1 are oxidized, changing KEAP1 conformation. This conformational change affects the interaction between NRF2 and KEAP1 inhibiting NRF2 ubiquitination and degradation. In normal cells TP53 is maintained at low levels by interaction with the negative regulator MDM2 a p53 highly specific ubiquitin ligase. H_2_O_2_ mainly regulates MDM2 activity by activating the ATM and c-ABL kinases involved in MDM2 phosphorylation. MDM2 phosphorylation inhibits its ubiquitination activity and stabilizes TP53 **(C)**. The MDM2 ubiquitin ligase substrate preference for TP53 is enhanced by MDMX. Phosphorylation of MDMX in different residues by c-ABL, AKT and CHK2 kinases inhibit TP53 degradation, while those catalyzed by the CK1α kinase stimulates TP53 degradation. All the referred kinases activities are regulated by H_2_O_2_. The association between the TF and the ubiquitination machinery can also be modulated by PTMs of the transcription factor **(D)**. HIF-1α is tagged with ubiquitin for degradation after being hydroxylated by PHD in the presence of O_2_ (D). H_2_O_2_ inhibits PHD that leads to HIF-1α stabilization. Factors colored blue are inhibitors of TF-dependent gene expression; factors colored red are activators of TF-dependent gene expression. Dashed lines indicate activation/inhibition.

Besides the regulation of the general proteasome catalytic activity [185], H_2_O_2_ is able to modulate the degradation of specific proteins mainly by two mechanisms. In the first mechanism H_2_O_2_ targets the E3 ligase complex, as are the cases for NRF2 and TP53, and in the second, PTM of the TF either increases (acetylation of AP-1 protein Jra) or decreases (*e.g.* c-JUN and NRF2 phosphorylation and HIF-1 hydroxylation) its association with the E3 ligase complex (Fig. 6A).

#### Targeting the E3-complex

##### Direct targeting

The best known example by which H_2_O_2_ targets the E3-complex involves the factor Kelch-like ECH-associated protein 1 (KEAP1). KEAP1 serves as the substrate adaptor subunit in the E3 holoenzyme in the ubiquitination pathway, leading to NRF2 ubiquitination and degradation [186,187]. In the presence of either electrophilic agents or reactive oxygen species critical cysteine residues in KEAP1 are alkylated or oxidized, leading to a conformational change of KEAP1, due probably to its zinc-finger nature. Such conformational alterations inhibit the binding between NRF2 and KEAP1, thus stopping NRF2 ubiquitination and degradation. There has been an ongoing discussion in the literature whether NRF2 dissociates from oxidized KEAP1 [22]. A recent report based on quantitative fluorescence recovery after photobleaching indicates that such dissociation does not occur [188], and so the NRF2 molecules that translocate to the nucleus are those synthesized *de novo* (Fig. 6B). Human KEAP1 contains 27 cysteine residues, and different agents modify different cysteine residues that translate into specific biological effects [92]. Concerning H_2_O_2_, in HeLa cells, Cys151 is the critical sensor residue that mediates the formation of an intermolecular disulfide – Cys151–Cys151 – between two KEAP1 molecules [189]. A mutation of this residue impairs NRF2 stabilization in the presence of H_2_O_2_. In addition, an intramolecular disulfide bond between Cys226 and Cys613 is promoted by H_2_O_2_, but mutants impairing the formation of this disulfide bond do not show functional alterations [189]. Unfortunately there is no available data concerning the reactivity between KEAP1 cysteine residues and H_2_O_2_. Based on the data of Fourquet et al. [189], we estimated that the rate constant for this reaction is at 140 M^−1^ s^−1^ (Fig. 3B). There are many assumptions in this estimate, but it is safe to say that cysteine residues in KEAP1 have a relatively low reactivity with H_2_O_2_.

##### Mediated targeting

Like it happens for NRF2, the cellular TP53 levels are mainly regulated by its ubiquitin-mediated proteasomal degradation [190,191]. In normal cells TP53 is maintained at low levels by interaction with the negative regulator MDM2 [192] a ubiquitin ligase E3 with high specificity for TP53 [[193], [194], [195]]. Regulation of MDM2 activity by H_2_O_2_ is largely done *via* PTMs, mainly phosphorylation, that by inhibiting its ubiquitination activity stabilize TP53 (Fig. 6C). The H_2_O_2_-activated kinases Ataxia telangiectasia mutated (ATM) [196] and c-ABL [197] phosphorylate MDM2 leading to the fast activation of TP53 [198,199].

A PTM that stabilizes MDM2 and promotes its ability to ubiquitinate TP53 is sumoylation [200]. Sumoylation of MDM2 is downregulated by H_2_O_2_ through the formation of disulfide bonds between the catalytic cysteine residues of the SUMO E1 subunit Uba2 and the E2-conjugating enzyme Ubc9 [201,202].

The protein MDMX is an additional key partner to the couple TP53/MDM2 and a potential target for H_2_O_2_. MDMX is essential for the MDM2-mediated TP53 polyubiquitination [203] as it enhances MDM2 substrate preference towards TP53 [204]. Phosphorylation of MDMX at two residues, Tyr99, catalyzed by c-ABL [205] and Ser367, catalyzed by either AKT [206] or CHK2 [207], activates TP53 by decreasing its MDM2-dependent ubiquitination (Fig. 6C). These PTMs are potentially stimulated by H_2_O_2_ because H_2_O_2_ activates the kinases c-ABL [197], AKT [24] and CHK2 [208]. On the other hand, phosphorylation of MDMX at Ser289 catalyzed by casein kinase 1 alpha (CK1α), stimulates the binding between MDMX and TP53 and leads to an inhibition of TP53 activity. Low levels of H_2_O_2_ promote the rapid dephosphorylation of CK1αLS, a nuclear splice form of CK1α, and enhances the kinase activity [209] (Fig. 6C).

#### Posttranslational modification of the transcription factor

A more common mechanism by which H_2_O_2_ stabilizes TFs is, perhaps, mediated by PTMs that modulate the association between the TF and the ubiquitination machinery. Here we describe a few cases involving different PTM of the TFs.

##### Phosphorylation

NRF2 can be tagged for degradation by a KEAP1-independent mechanism that is controlled by glycogen synthase kinase 3β (GSK-3β) [210]. Active GSK-3β catalyzes the phosphorylation of NRF2 in the Neh6 domain, forming a phosphodegron that is recognized by the substrate receptor β-transducin repeat-containing protein (β-TrCP) complex, followed by NRF2 ubiquitination and degradation [211]. GSK-3β is inhibited by phosphorylation of Ser9 catalyzed by AKT kinase [212]. Thus, H_2_O_2_, being an activator of the PI3K/AKT pathway, inhibits GSK-3β [213,214] activating NRF2 in a KEAP1-independent manner. In fact, inhibition of the PI3K/AKT pathway by LY294002 has been shown to block partially the activation of NRF2 by H_2_O_2_ [215]. Other kinases that inactivate GSK-3β are ERK, p38 MAPK and PKC [216], and it has been suggested that these kinases may regulate NRF2 indirectly by inhibiting GSK-3 [217]. All of these kinases are known to be activated by H_2_O_2_, and so they may also mediate H_2_O_2_ regulation of NRF2 [[218], [219], [220]]. It should be mentioned that a prolonged exposure to high H_2_O_2_ concentrations with concomitant cell toxicity activates GSK-3β [215,221,222], which could be an NRF2 termination signal.

Two additional mechanisms that involve NRF2 phosphorylation causing dissociation of the NRF2/KEAP1 complex, NRF2 stabilization and, ultimately, leading to its nuclear translocation have been identified:

• Phosphorylation catalyzed by protein kinase RNA (PKR)-like ER kinase (PERK), a transmembrane protein kinase that is required for the cellular response to ER stress [223], and that is rapidly activated (15 min) by low extracellular H_2_O_2_ levels (15 μM) in HeLa cells [224].
• Phosphorylation at Ser40 catalyzed by PKC-δ [225], a kinase that is well known to be activated by H_2_O_2_.

H_2_O_2_ also controls the poly-ubiquitination of the AP-1 members c-JUN and CREB by modulating their PTMs [226,227]. c-JUN phosphorylation, catalyzed either by JNK, at the two clusters Ser63/Ser73 and Thr91/Thr93 [226], or by c-ABL kinase at Tyr170 [228] stabilizes c-JUN, by decreasing c-JUN ubiquitination and proteasomal degradation. Thus H_2_O_2_ by activating both the apoptosis signal-regulating kinase 1 (ASK1), which activates JNK (see section Modulation of DNA binding by PTM), and c-ABL [197] kinases, may stabilize c-JUN. However, there are conflicting reports concerning the role of Tyr170 in c-JUN stabilization [229]. Concerning CREB, a long-term treatment with a low concentration of H_2_O_2_ increases CREB-Ser133 phosphorylation and decreases CREB protein abundance *via* a proteasomal mechanism, but Ser133 phosphorylation is not necessary for CREB degradation [227]. It is possible that either PKD1 (protein kinase D1) regulates CREB phosphorylation at a site other than Ser133 in cardiomyocytes or that the lower levels of CREB are due to the phosphorylation of a different cellular PKD1 substrate able to regulate CREB protein levels indirectly [227].

##### Hydroxylation

Hydroxylation plays a key role controlling HIF activation during hypoxia by increasing HIF-1α protein degradation. In this system, PHD uses O_2_ to catalyze the hydroxylation of the sub-unit HIF-1α, which is subsequently tagged with ubiquitin for degradation [165,230]. However, the regulatory mechanism leading to HIF-1 activation and stabilization was shown to be more complex than a simple O_2_ sensing mechanism. Many other factors were shown to play a role in the regulation of HIF-1α expression, particularly reactive oxygen species, which have been the center of intense investigation. The molecular mechanism underlining H_2_O_2_ effect in HIF stabilization has been attributed mainly to an inhibition of PHD activity (Fig. 6D). It has been shown that an increase in the amounts of H_2_O_2_ due to glucose oxidase addition to the cell medium inhibits hydroxylation of HIF-1α [231]. Although HIF-1α has several cysteine residues, their oxidation has never been observed. However, it has been proposed that H_2_O_2_ is able to oxidize Fe^2+^, present in the catalytic site of PHD, to Fe^3+^ by a Fenton reaction, inactivating the enzyme [232].

In hypoxic conditions, HIF-1α protein stability and downstream gene target activation were shown to be increased by overexpression of NOX1 [233]. Furthermore, the high levels of mitochondria-derived reactive oxygen species generated by hypoxia were considered to be essential for HIF-1α stabilization and this effect was reversed upon overexpression of either catalase or glutathione peroxidase 1 [134,234,235]. However, the role of H_2_O_2_ in HIF-1α stabilization in hypoxic conditions is still unclear. Recent data has shown that thioredoxin 1 overexpression, although able to block the increase in reactive oxygen species levels under hypoxia, has neither an effect on the levels of HIF-1α nor in the activation of its downstream targets [236]. Besides H_2_O_2_, superoxide radical also increases HIF-1α stabilization and might function as an alternative mechanism [135].

H_2_O_2_ has also been shown to be an HIF-1 regulator in normoxia. Besides hypoxia, HIF-1 is also upregulated in response to various growth factors, cytokines and hormones in normoxia [237]. The activation of these signaling pathways induces the production of H_2_O_2_ dependent on the activation of NADPH oxidases that induce HIF-1α stabilization [238,239].

##### Acetylation

Ubiquitination of Jra, a *Drosophila* Jun protein is facilitated by its acetylation, in a process that is regulated by both Cbp and Sir2 *in vivo* [240]. Because protein acetylation is regulated by H_2_O_2_ [241], it may be hypothesized that this constitutes another potential pathway for activation of AP-1 by H_2_O_2_.

### Cytoplasm-nuclear trafficking

**Fig. 7:**
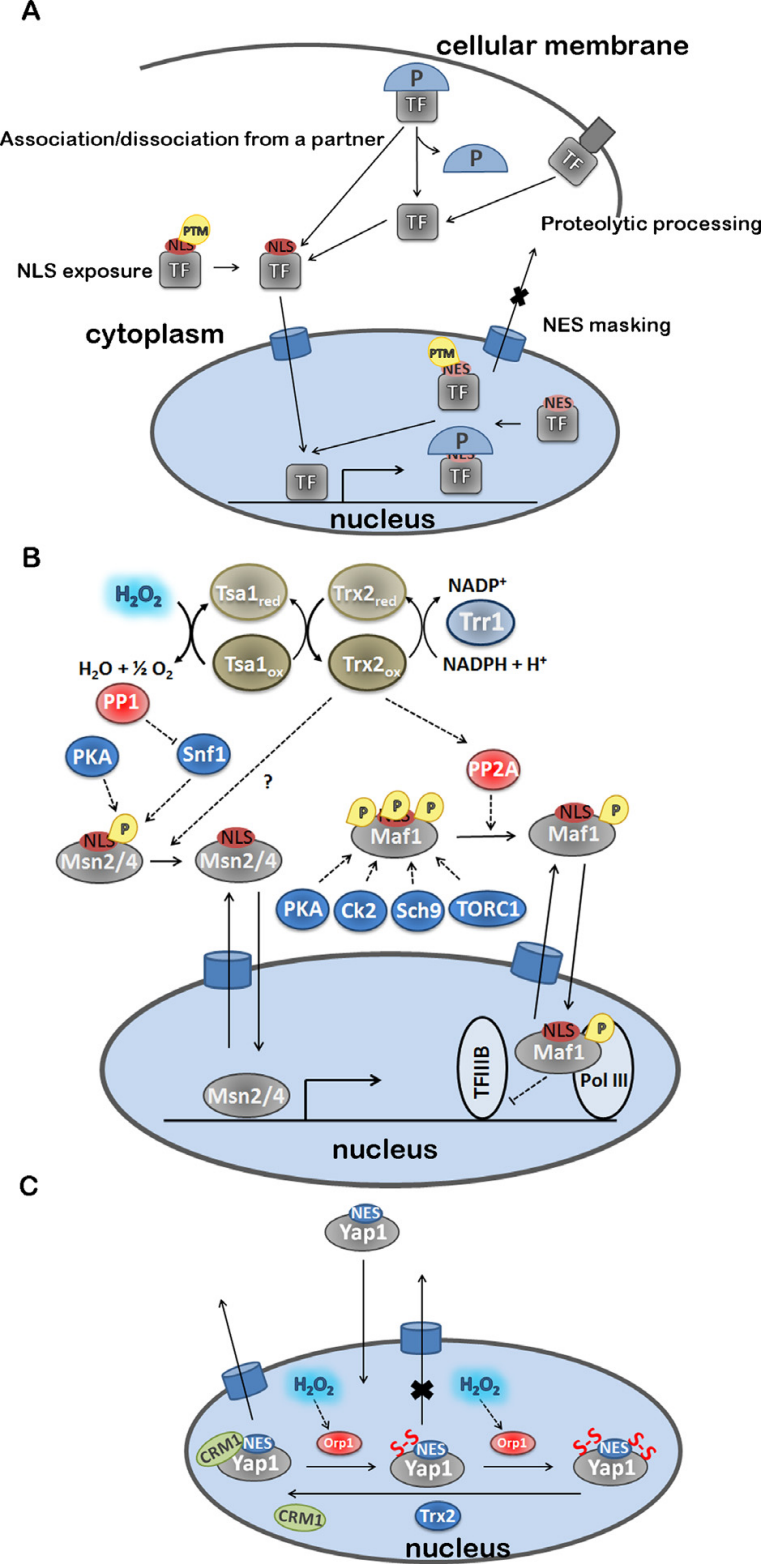
Regulation of cytoplasm-nuclear trafficking of TF by H_2_O_2_. **(A)** Nuclear localization of TF is essential for gene expression activation and H_2_O_2_ plays a key role in TF cellular trafficking. H_2_O_2_ modulates NLS exposure by removing PTM or by promoting partner dissociation (P). In certain cases, the association to adaptor proteins might promote NLS exposure. Inversely, NES masking is another mechanism to retain TF in the nucleus that is mediated by H_2_O_2_. Conformational changes induced by PTM together with the formation of protein complexes make NES inaccessible inducing activation of transcription. Other TF are associated to cellular membranes in their inactive state. The activation of these TF requires proteolytic cleavage and release to the cytoplasm where it is transported to the nucleus. **(B)** Msn2/4 and Maf1 translocation to the nucleus is activated after dephosphorylation, which uncovers NLS. This process is activated by H_2_O_2_ indirectly through Trx system. Msn2/4 and Maf1 dephosphorylation is dependent upon Trx2 by an unknown mechanism and by PP2A activation, respectively. **(C)** Yap1-dependent gene activation depends on its retention in the nucleus by CRM1 dissociation that occurs in presence of H_2_O_2_. The oxidation of four Cys residues in Yap1 is responsible for the conformational alterations that prevent NES recognition by CRM1. As for Msn2/4 and Maf1, Yap1 does not react directly with H_2_O_2_, and its oxidation is mediated by a GPx, Orp1. Trx2 reduces Yap1 inducing its translocation to the cytoplasm, inactivating gene transcription. Factors colored blue are inhibitors of TF-dependent gene expression; factors colored red are activators of TF-dependent gene expression. Dashed lines indicate activation/inhibition.

If the inactive form of a TF rests in the cytoplasm, after activation the TF must be transported from the cytoplasmic pool to the nucleus, were it can affect the transcription of the target genes. There are a few mechanisms by which the accumulation of a TF can be achieved upon exposure to H_2_O_2_: PTM in the TF itself that either exposes an NLS (mammalian HSF1, Maf1, and Msn2/4) or masks a NES (Yap1, and NRF2); release/association from/with a partner (NFκB/IκB, NRF2/KEAP1, and TP53/MDM2). In other cases the TFs precursors are kept in the cytosol associated to membrane anchors; for their activation to occur these factors must be processed, which enables the TFs to enter the nucleus, where the nuclear form activates target genes (SREBP and NOTCH). Below we describe in detail each of these cases where H_2_O_2_ activates redox-dependent TFs (Fig. 7A).

#### Activation mediated by post-translational modification in the transcription factor

##### NLS exposure

Mammalian HSF1 provides an interesting example in which trimerization of the TF mediates exposure of an NLS. In non-stressed situations, this TF exists as a monomer and, upon exposure to H_2_O_2_ forms a trimer, in a reaction involving the formation of disulfide bonds [242]. Redox-regulation of HSF1 multimerization, and its nuclear accumulation are early and linked steps in HSF1 activation. In fact, the mutants HSF1 Cys35Ser, HSF1 Cys105Ser and HSF1 Cys35105Ser, which are defective in stress-induced multimerization *in vivo* and *in vitro*, are also defective for stress-induced nuclear accumulation [242].

Two yeast TFs that in resting conditions are mainly present in the cytosol and whose H_2_O_2_-dependent activation is mediated by thioredoxins are Maf1 and Msn2/4. Both TFs are controlled by their phosphorylation state and, upon activation, undergo dephosphorylation and translocation to the nucleus. In both cases, rapid nuclear translocation and TF activity are impaired in *trx1*Δ*trx2*Δ cells lacking cytosolic thioredoxins upon exposure to 0.1–0.8 mM extracellular H_2_O_2_ [243] (Fig. 7B).

The negative regulator Maf1 represses RNA polymerase III activity under carbon source starvation, ER stress (5 mM DTT), and oxidative stress (0.5 mM extracellular H_2_O_2_) conditions. Maf1 phosphorylation, some of which occurs in the vicinity of an NLS, is mediated by Sch 9 [244], c-AMP-dependent protein kinase A (PKA) [245,246], TORC1 [247] and casein kinase II (CK2) [79]. The cAMP–PKA system is not a key regulator of the H_2_O_2_-dependent Maf1 nuclear translocation in yeast [243], and it is not known whether the activities of other kinases acting on Maf1 (Sch 9, TORC1 and CK2) are decreased by H_2_O_2_. What has been established so far is that the activity of protein phosphatase 2A (PP2A) is essential for Maf1 dephosphorylation and nuclear accumulation in cells treated with H_2_O_2_ [243,248] . This pathway is regulated by oxidized cytosolic thioredoxins because in *trx1*Δ*trx2*Δ cells treated with 0.8 mM extracellular H_2_O_2_, Maf1 remains in the cytosol and its dephosphorylation is impaired [243] (Fig. 7B).

Msn2 is a direct substrate of protein kinases PKA [245] and Snf1 [249], and most of Msn2 resides in the cytosol when phosphorylated in its NLS. Dephosphorylation of Msn2 may occur due to downregulation of kinases but the rapid dephosphorylation of Msn2 under glucose depletion suggests that protein phosphatases must also play an important role in the activation of this TF [249], namely PP1 [249] and PP2A [250]. However, contrary to what happens in other stress conditions, for H_2_O_2_-induced Msn2 nuclear translocation there is no correlation with a decreased PKA activity and translocation is not dependent on PP2A activity [243]. Therefore, the exact mechanism of Msn2/4 activation by H_2_O_2_ is still unknown, although, like with Maf1 is mediated by thioredoxins. H_2_O_2_-induced Msn2/4p nuclear localization is not only impaired in *trx1*Δ*trx2*Δ cells but also in *trr1*Δ*trx1*Δ*trx2*Δ cells, which besides lacking cytosolic thioredoxins also have no thioredoxin reductase, and in *tsa1*Δ cells, which lack the most abundant of the yeast Prxs [243]. However, the levels of nuclear Msn2 when H_2_O_2_ (0.1 mM, 10 min) is added to *trr1*Δ cells, which only lack thioredoxin reductase, are higher than in wild-type cells [243]. These results suggest that H_2_O_2_-dependent activation of Msn2 involves reaction of H_2_O_2_ with Tsa1 which is then reduced by the thioredoxin system forming oxidized thioredoxins. How the oxidized thioredoxins transmit the signal to Msn2/4 has not yet been established. However, site directed mutagenesis of the unique Cys residue present in a truncated Msn2 that still exhibits the behavior of the full protein established that there was still thioredoxin-dependent translocation to the nucleus [243]. Therefore, H_2_O_2_-dependent Msn2 translocation does not involve a direct oxidation of a cysteine residue by the oxidized thioredoxins.

##### NES masking

Yap1 and NRF2 are two TFs that undergo nuclear export mediated by CRM1 (chromosome maintenance region 1). For Yap1 this export is absolutely central for the control of its activation by H_2_O_2_ and we will describe the process in detail.

Yap1 is found in the cytoplasm under non-stressed conditions but rapidly accumulates in the nucleus following oxidant exposure [75,251]. The cytoplasmic localization is determined by constitutive nuclear export that predominates over constitutive nuclear import [75,252,253]. The activation of Yap1 by H_2_O_2_ requires the multistep formation of disulfide bonds in the CRD and/or the cCRD domains [254]. The intramolecular disulfide bond between Cys303 (cCRD) and Cys598 (nCRD) formed in Yap1 after H_2_O_2_ exposure masks the NES present in the cCRD domain, resulting in structural changes in the NES [76]. This allows the Yap1–Crm1 complex to be disrupted and Yap1 accumulates in the nucleus [252,255,251]. It is important to note that, depending on the oxidative stress inducing agent, Yap1 can undergo two distinct conformational changes, both involving disulfide bond formation, and both masking the nuclear export signal, thus abolishing nuclear export by Crm1 [76]. In the case of H_2_O_2_ cysteine residues in Yap1 are not oxidized by a direct reaction with this agent but require GPx3/Orp1, which acts as sensor protein for H_2_O_2_ [256]. GPx3/Orp1 has *in vitro* peroxidase activity by a mechanism involving a Cys36–Cys82 catalytic disulfide, which is distinct from those of classical glutathione peroxidases. Also, Orp1 is recycled by thioredoxin and not by GSH. Gpx3/Orp1 becomes oxidized at Cys36 which forms a heterodimeric disulfide bond with Cys598 in Yap1 upon H_2_O_2_ exposure [256]. Two more thiol/disulfide exchange reactions finally result in oxidized Yap1, with formation of a disulfide bond between Cys303 and Cys598, and reduced GPx3/Orp1. The second disulfide bond in Yap1 (Cys310–Cys629) presumably forms through a similar pathway on recruitment of another oxidized GPx3/Orp1 molecule, only after the first disulfide bond has formed [257]. GPx3/Orp1 is reduced by the thioredoxin/thioredoxin reductase system [256]. Yap1 nuclear export is restored when disulfide bonds are reduced by Trx2, whose expression is controlled by Yap1, providing a mechanism for negative autoregulation [256,251]. Besides GPx3/Orp1, there is another protein, Ybp1, which forms a stress-induced complex with Yap1 *in vivo* and influences the nuclear localization of Yap1 in response to H_2_O_2_ [258,259]. It has been proposed that the role of Ybp1 might be to increase the efficiency of this process *in vivo* to more rapidly prepare a defensive response to an H_2_O_2_ challenge (see Fig. 7C).

Concerning NRF2 export from the nucleus, NRF2 possesses both a redox-sensitive [260] and a redox-insensitive [261] NES but, ultimately, both mediate H_2_O_2_ regulation. The redox-sensitive site is present in the Neh5 transactivation domain of NRF2, where the redox state of Cys183 has a critical role. Mutation of this residue abolishes the rapid activation of NRF2 by 50 μM extracellular H_2_O_2_ in HeLa cells, which led to the conclusion that oxidation of Cys183 inhibits binding of NRF2 to the nuclear exporting protein CRM1 [260]. The redox-insensitive NES is present in the leucine zipper (ZIP) domain of NRF2, and phosphorylation of Tyr568 residue present in its vicinity, strengthens interaction with CRM1 to enhance nuclear export of NRF2 [261,262]. The kinase responsible for this PTM is FYN, which is activated by GSK-3β. [221]. Thus, prolonged exposure to high H_2_O_2_ concentrations, with concomitant cell toxicity [222], activates GSK-3β, induces NRF2 phosphorylation at Tyr568 and NRF2 degradation [215,221]. On the other hand, short-time incubation with H_2_O_2_ inhibits GSK-3β [213,214], activating NRF2. It has been proposed that phosphorylation at Tyr 568 may mediate a failed antioxidant response, leading to cellular toxicity, and eventually cell death [215] or, alternatively, it may constitute a switch-off mechanism [221]. An additional mechanism by which NRF2 export is controlled is by its binding to small MAF bZIP proteins, which enhances nuclear retention of NRF2 by masking the NES motif in the NRF2 ZIP domain [90].

#### Activation mediated by association/dissociation from a partner

NF-κB is a TF whose central activation step involves its release from an inhibitory protein, IκB, that masks an NLS in NF-κB. Concerning the most abundant form of NF-κB, the dimer p65–p50, H_2_O_2_ is considered a modulator of activation induced by other agents, namely cytokines, and not an inducer *per se* [22,115,263]. Nevertheless, recent evidence indicates that in MCF-7 cells H_2_O_2_ (25 μM steady-state) induces c-REL nuclear translocation to values similar to those obtained with physiological levels of TNF-α [42]. The mechanisms by which H_2_O_2_ promotes degradation of IκB, thus exposing the NLS of NF-κB and subsequent NF-κB activation are complex and are not well defined. IκB is signaled for proteasome degradation following its phosphorylation catalyzed by the IKKs. The IKK complex consists of a dimer of IKKγ subunits linked through a disulfide bond forming the NF-κB essential modulator (NEMO) to which IKKα and IKKβ bind in the resting state. Activation of IKK is made by phosphorylation catalyzed by an IKK kinase, either TGFβ-activated kinase (TAK1) or AKT, in the canonical pathway of activation, or NF-κB inducing kinase-1 (NIK1) in the alternative pathway, which can all be modulated by H_2_O_2_ [115,22]. Alternatively, LC8, a substrate of the redoxin TRP 14, may mediate H_2_O_2_ effects. In the reduced form LC8 binds to IκB and inhibits its phosphorylation by IKKs and subsequent degradation, and thus H_2_O_2_ by forming LC8 dimers linked *via* a disulfide bond [264], promotes LC8 dissociation from IκB, and NF-κB activation [265]. On the other hand, inhibition of activation of NF-κB by H_2_O_2_ could be mediated by KEAP1-dependent degradation of IKKβ [266].

Concerning NRF2, in addition to the mechanism previously described, its cellular localization is also mediated by interaction with its partner, KEAP1. NRF2 contains three NLS, one of which is present in the NEH2 domain of NRF2, the domain that mediates interaction of NRF2 with KEAP1 [267,268]. So, NRF2 that is not bound to KEAP1 tends to translocate to the nucleus [269]. NRF2 can also be exported from the nucleus bound to KEAP1, which binds to CRM1 through a NES [270], being subsequently degraded in the cytosol [271]. KEAP1 does translocate to the nucleus by an unknown mechanism [272], possibly by inhibition of its export from the nucleus. It has been observed that both mutation of KEAP1 Tyr85 [273] and oxidative modification within an NES of nuclear KEAP1 blocks NRF2-KEAP1 complex export from the nucleus [270].

In the case of TP53, there are several evidences showing that H_2_O_2_ promotes TP53 nuclear localization [157,274] but the mechanisms involved are not clearly established. The MDM2 protein, which acts in conjunction with CRM1, mainly regulates the TP53 nuclear export through NES [108,275]. Consequently, factors that regulate the specific cellular compartment localization of MDM2 also control TP53 cellular localization. For example, the p14ARF protein causes MDM2 nucleolus permanence, and c-Abl binds to MDM2 preventing its interaction with TP53, both leading to nuclear retention of TP53 [199,276,277]. On the other hand the phosphorylation of MDM2 through the PI3K/AKT pathway promotes MDM2 nuclear import, resulting in TP53 nuclear export [[278], [279], [280]]. Interestingly, HSF1 is required for TP53 nuclear importation and activation, which implies that heat shock factors play a role in the regulation of TP53. As already mentioned MDM2 is regulated by H_2_O_2_ and, therefore, the nuclear localization of TP53 induced by H_2_O_2_ may be mediated by MDM2.

The cytoskeleton may also play an important role as a partner in TP53 translocation. TP53 phosphorylated forms, namely at Ser15, show increased binding to microtubules and a lower tendency to nuclear translocation after exposure to chemotherapeutic agents [281]. It is conceivable that H_2_O_2_ may affect TP53 microtubule binding and nuclear translocation through alterations on TP53 phosphorylation status since several reports describe the phosphorylation of Ser15 in TP53 upon H_2_O_2_ exposure (see Table 2).

**Table 2:** Phosphorylated and acetylated TP53 amino acid residues in different cell types exposed to extracellular single doses of H_2_O_2_.

Cell type	[H_2_O_2_] (µM)	Exposure time	Domain	Phosphorylation	Kinases	Biological role	References
GM00637	200		N-terminal domain (transactivation)	Ser9, Ser15, Ser20	Ser20 residue requires Plk3 activated by ATM	Induction of p21, suggesting functional activation of p53	[334]
A549	50, 100	30 min	C-terminal domain (regulatory)	Ser15, Ser20, Ser37 or Ser392	ATM-dependent except for Ser37 and Ser392		[368]
HUVEC	100	5 min to 1 h	N-terminal domain (transactivation)	specifically at Ser15	ATM kinase (downstream PDGF receptor)	Increased transcription of p21^CIP/WAF^ gene	[333]
NHF	100	1 h	N-terminal domain (transactivation)	Thr81	JNK	P53 stabilization and activation of transcriptional activities	[369]
Human keratinocytes	50, 100	1 h or 16 h	N-terminal domain (transactivation)	Ser15?	AMPK?	Senescence in human keratinocytes by activating p21^CIP1^	[370]
AF5	400	2 h, 6 h, 12 h	N-terminal domain (transactivation)	Ser15	?	Induction of P21, MDM2 and apoptotic proteins	[160]

				**Acetylation**	**Deacetylases**		
TIG-3	150	1 h	C-terminal domain (regulatory)	Lys373 and 382	Downregulation of SIRT1	Cell senescence; induction of p21	[344]
HUVEC	0, 25, 50 100	6 days	C-terminal domain (regulatory)	Lys382	Downregulation of SIRT1	Unaltered levels of P53, P21, Bcl-2, and Bax; Up-regulation of p16INK4a: G1 arrest and aging	[371]
Human skin keratinocytes	50, 125,250	0.5 and 2 h	C-terminal domain (Regulatory)	Lys382	Downregulation of SIRT1 involving JNK activation	Induced cell death	[343]

#### Activation through release from membrane by proteolytic processing

For both NOTCH and SREBP1c the critical activating event is their proteolytic processing and release from the plasma membrane and the ER, respectively. These proteolytic events may be controlled by H_2_O_2_, activating NOTCH signaling and having cell-type dependent effects for SREBP1c.

In the case of NOTCH signaling, it was observed that colonic tissues of NOX1^KO^ mice present less NICD compared to wild-type animals and a consequent decrease in the activation of NOTCH target genes [282]. The increase of NICD proteolysis by H_2_O_2_ was also observed for NOTCH3 in human aortic endothelial cells treated with extracellular H_2_O_2_ (50 µM, 1 h) or, inversely, with extracellular catalase added to the cultures [283]. These authors also showed a concomitant decrease in NECD in the presence of H_2_O_2_. ADAM17/TACE, the metalloproteinase responsible for the NECD release, has been demonstrated to be activated by H_2_O_2_ through the oxidation of critical sulfhydryls of the protein, suggesting its involvement in the redox regulation of NOTCH signaling [284,285].

Concerning SREBP1c, this TF is predominantly produced in the liver and white adipocytes, and controls fatty acid biosynthesis under glucose/insulin stimulation [138,286]. In HepG2 cells, extracellular H_2_O_2_ (100, 250, and 500 µM for 3 h) stimulates expression of SREBP-1 gene as well as its target genes involved in lipid accumulation [139], while in COS-7 cells H_2_O_2_ suppresses SREBP-1c transcriptional activity. The stimulatory effect of H_2_O_2_ may be mediated by the insulin signaling pathway at least in part through the oxidative inhibition of protein tyrosine phosphatases [286]. It is also possible that H_2_O_2_ activates SREBP by inducing ER stress [287], producing the mature forms of the SREBP-1c that translocate to the nucleus. It is important to note the different response to H_2_O_2_ observed between the two types of cells, HepG2 and COS-7 cells. Such differences may be due to a difference in nutrient metabolic function, which could cause differences in SREBP-1c needs, highlighting the specialized role of this TF.

### DNA binding and transactivation

**Fig. 8:**
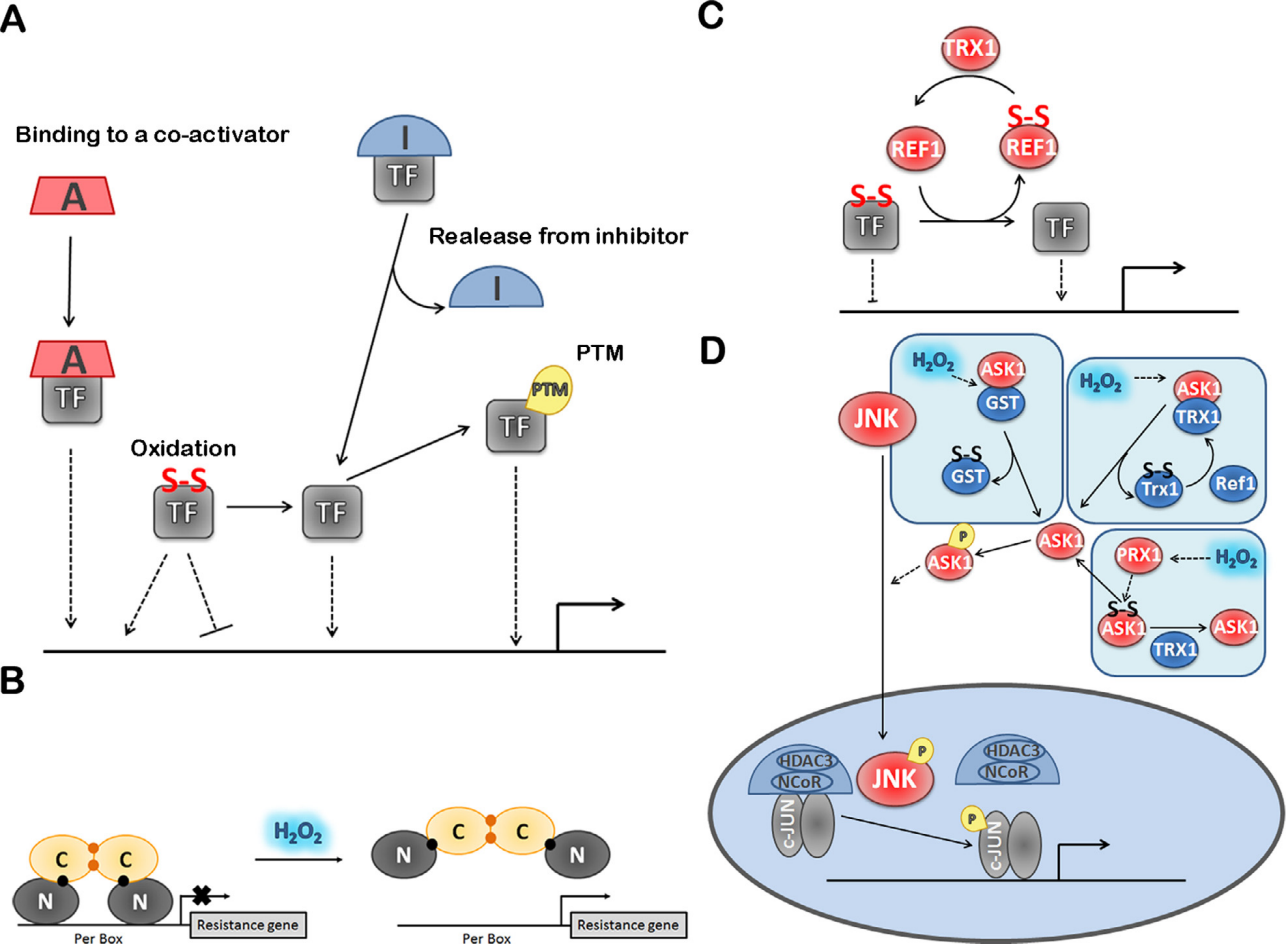
Regulation of DNA binding and transactivation of TF by H_2_O_2_. **(A)** TF affinity to DNA or to transcription activators is regulated by H_2_O_2_ through different mechanisms involving either conformational alterations induced by PTM or association with activators/inhibitors. In addition, H_2_O_2_ is able to modulate TF activation by direct TF oxidation inside or outside DNA binding domain. **(B)** PerR DNA affinity is lost by a conformational alteration induced by a Fenton reaction of H_2_O_2_ with Fe^2+^ forming 2-oxo-histidine. Both Fe2+and Mn^2+^ (black spheres) can bind to the regulatory site but when Mn^2+^ is bound 2-oxo-histidine is not formed. Structural ZnII ions (yellow spheres). **(C)** Ref-1 induces the DNA binding activity of several TFs by reducing cysteine residues of those TFs. Ref-1 is regenerated by thioredoxin that reduces Cys65. **(D)** H_2_O_2_ promotes c-Jun phosphorylation releasing its repressor complex, which contains HDAC3. This phosphorylation is dependent of the activity of JNK, which is activated by ASK1. ASK1 activation is regulated by H_2_O_2_ through three alternative mechanisms: GST oxidation releasing ASK1, Trx oxidation releasing ASK1 or ASK1 oxidation by PRX1. Factors colored blue are inhibitors of TF-dependent gene expression; factors colored red are activators of TF-dependent gene expression. Dashed lines indicate activation/inhibition.

In the chain of events we have been describing transactivation and DNA binding represent the last step in order for a gene to be activated by a TF. In the simplest case, the TF senses directly H_2_O_2_, changes its conformation, and as such it increases (*e.g.* OxyR and mammalian HSF1) or decreases (PerR) its affinity towards DNA. Many mammalian TFs contain cysteine residues in their DNA binding domain whose oxidation impairs DNA binding, constituting an additional layer of control in a complex signaling pathway. In addition, H_2_O_2_ modulation mediated by PTM of the TF can modulate the affinity of the TF either towards DNA (c-JUN, SP-1, CREB, and NRF2), towards a co-activator (HIF-1) or towards a repressor (c-JUN, Yeast Hsf). The state of chromatin adds another layer of control and, actually, H_2_O_2_ is able to target specific regions of chromatin to selectively activate individual genes, as exemplified for SP1 (Fig. 8A).

#### Modulation of DNA binding by oxidation or reduction

##### Oxidation of the transcription factor

In bacterial TFs and in the TF responsible for mammalian general stress response, HSF1, the signaling pathway boils down to controlling binding/dissociation from DNA. In these examples the amino acid residues or prosthetic groups that react with H_2_O_2_ mediate affinity towards DNA.

###### Cysteine oxidation.

In *E. coli*, OxyR functions as a sensor of H_2_O_2_ and a transducer of oxidative stress due to its capacity to be oxidized directly [61,67]. Different studies have been done in order to understand the mechanism underlying oxidant sensing by OxyR (see review [288]). Two basic models have been proposed: (i) in the first model, reduced OxyR reacts directly with H_2_O_2_ to form a sulfenic acid at Cys199, which rapidly forms a disulfide bond with Cys208. The formation of this specific disulfide bond induces structural changes in the regulatory domain leading to altered DNA binding properties and allowing a productive interaction between OxyR and RNA polymerase, which then triggers expression of the target genes. OxyR is rapidly deactivated by slow reduction *via* a glutathione-dependent mechanism [23,[289], [290], [291]; (ii) in the second model, the “molecular code” hypothesis, modification of Cys199 alone is sufficient to activate OxyR in the absence of disulfide bond formation; several PTMs can occur in Cys199, including sulfenylation (Cys199-SOH), *S*-nitrosylation (Cys199-SNO), and *S*-glutathionylation (Cys199-S-S-G) forming a modified OxyR able to activate the transcription of target genes [292]; although all these modifications lead to a transcriptionally active OxyR, they differ in structure, cooperative properties, DNA binding affinity, and promoter activities. By this second mechanism, the authors suggest a redox “molecular code” resulting in a more selective regulatory response in which distinct subsets of OxyR target genes are differentially regulated depending on the particular oxidized form of OxyR involved. Recent data shows that Cys199 nitrosylation is associated with the activation of a sub-set of genes that differ from those upregulated in response to H_2_O_2_ [293], which supports the “molecular code” mechanism. Probably both mechanisms operate *in vivo* [67].

Mammalian HSF1 can be activated directly by H_2_O_2_ [242]. The initial step in HSF1 activation is its trimerization. The trimerization of HSF1 increases the affinity of HSF1 for HSEs by several orders of magnitude [294]. Multimerization of monomeric recombinant mouse Hsf1 and Hsf1 DNA binding is induced *in vitro* by heat shock temperatures and by extracellular H_2_O_2_ (0.1 µM–200 µM) [242]. Ahn and Thiele [242] were able to show, using site directed mutagenesis, that *in vivo* activation of Hsf1 by extracellular H_2_O_2_ (500 µM, 1 h) requires the formation of a disulfide bond between the two cysteine residues (Cys35 and Cys105) present in the Hsf1 DNA-binding domain. Similarly to what happens for murine Hsf1, H_2_O_2_, diamide and heat stress also induce the formation *in vitro* of an intramolecular disulfide bond between the Cys36 and Cys103 residues in the DNA-binding domain of human HSF1, which leads to HSF1 trimerization and DNA binding [295]. However, for human HSF1 it was found that another disulfide bond involves other cysteine residues (Cys153, Cys373 and Cys378) located in the trimerization and in the transcriptional activation domains, and their oxidation leads to inhibition of HSF1 trimerization and DNA binding [295].

###### Metal center oxidation.

PerR provides a very interesting example in which H_2_O_2_ sensing is mediated by Fenton chemistry and not cysteine oxidation. Because this is a less common mechanism for H_2_O_2_ signaling effects, we will discuss in some detail the mechanistic and structural aspects of the modification of this TF by H_2_O_2_. This TF is a transcription repressor and its oxidation releases it from the promoter regions of the target genes, thus activating gene expression. The crystallographic structures of PerR from *B. subtilis* [[296], [297], [298]] and *Streptococcus pyogenes* [299] have helped to gain insight into the mechanism of PerR as an H_2_O_2_ sensor and also of how Fe^2+^ binding to the protein leads to activation of PerR binding to DNA. Just like the other proteins of the Fur family, PerR is a homodimer and each monomer has two functional domains, an N-terminal DNA binding domain that contains a winged helix interaction motif and a C-terminal domain that is involved in the dimerization of the protein [296]. Each monomer also contains two metal-binding sites, a structural binding site with tightly bound Zn^2+^ coordinated by four cysteine residues [300] and a regulatory site, which under physiological conditions can be loosely occupied either by Fe^2+^ (PerR: Zn,Fe) or Mn^2+^ (PerR: Zn,Mn), and that is required for DNA binding [63]. The Zn(Cys)_4_ site gives structural stability to the dimer by locking together three β-strands present in the dimerization domain [66,296,300,301]. Insights into the structure of the regulatory site of PerR emerged both from mutational analyses [66] and from structural modeling based on the PerR apoprotein (PerR: Zn) [296]. Both studies suggest that the five ligands for the metal in the regulatory site, all conserved within PerR family members, consist of three histidine residues (His37, His91, and His93) and two aspartate residues (Asp85 and Asp104).

Lee and Helmann [300] estimated a rate constant of ˜10^5^ M^−1^ s^−1^ for the inactivation of PerR: Zn,Fe by H_2_O_2_, which is comparable to that for the oxidation of OxyR by H_2_O_2_ [291], and showed that PerR senses H_2_O_2_ at its regulatory site through metal-catalyzed histidine oxidation. The mechanism for PerR oxidation by H_2_O_2_ would rely on the production of a hydroxyl radical through the Fenton reaction, involving the bound Fe^2+^, leading to the rapid and direct incorporation of an oxygen atom into one of the histidine ligands (His37 or His91) forming 2-oxo-histidine [66]. The formation of 2-oxo-histidine has also been reported for the inactivation of Cu,Zn-superoxide dismutase [302] and in metal-catalyzed oxidations of histidine-containing enzymes [303]. Oxidation of His37 is the preferred site of 2-oxo-histidine formation and does not lead to changes in the affinity for the metal ion bound to the regulatory site. On the contrary, when His91 is oxidized PerR has a considerably reduced affinity for the regulatory site metal ion [297]. Oxidation of His37 and/or His91 in PerR prevents DNA binding due to a conformational change in PerR and the inactivated oxidized protein dissociates from the DNA, which leads to a derepression of the PerR regulon [66,297] (Fig. 8B). No repair mechanisms are known for 2-oxo-histidine, which has led to the proposal that oxidized PerR is not recycled [66].

The metal content of the regulatory site determines the ability of PerR to sense H_2_O_2_ [63,69,299]. While the PerR: Zn,Fe protein is highly sensitive to H_2_O_2_, peroxide induction of the PerR regulon through oxidation and inactivation of PerR is inefficient with the PerR: Zn,Mn protein. *In vivo* inactivation of PerR: Zn,Mn requires 10 mM extracellular H_2_O_2_, a level at least 1000 times higher than that needed for inactivation of PerR: Zn,Fe [300]. Fe^2+^ has an approximate 30-fold higher affinity for the regulatory site than Mn^2+^ and *in vivo* there is a competition between Fe^2+^ and Mn^2+^ for binding to the regulatory site [67]. Thus it is possible that *in vivo* PerR is regulated both by H_2_O_2_ levels and the relative levels of Fe^2+^ and Mn^2+^. So, when Fe^2+^ levels are low and Mn^2+^ levels are high the PerR regulon is not derepressed upon exposure to H_2_O_2_ [304].

##### Reduction of the transcription factor by Ref1 and thioredoxin

There are several TFs that contain cysteine residues that must be reduced in order to bind DNA, although *per se* this is not sufficient for TF activation. To what extent H_2_O_2_ oxidizes these cysteine residues *in vivo* to inhibit DNA binding is unknown. For example, the DNA-binding activity of SP1, which is strongly decreased by exposing nuclear protein extracts from 4-month-old rat tissues for 1 h with 5 mM–20 mM extracellular H_2_O_2_, is restored by the treatment with high dithiothreitol concentrations [305]. Concerning NF-κB, 1 mM extracellular H_2_O_2_, although able to induce NF-κB translocation to the nucleus in human endothelial cells, does not induce NF-κB-dependent transactivation of DNA [306].

Cells have a nuclear enzymatic system to keep cysteine residues in TFs reduced, the apurinic/apyrimidinic endonuclease/redox effector factor-1 (APE/REF-1) [307]. REF-1 was first recognized by its DNA base excision repair activity and, therefore, it was initially named apurinic/apyrymidinic endonuclease [308]. It is the rate limiting enzyme in the mammalian base excision repair pathway [309]. However, in addition to its DNA repair function, REF-1 also displays, in mammals but not in other vertebrates, a distinct redox regulation role as a redox co-activator of different TFs [307,310]. The two biological activities displayed by REF-1 are located in two functionally distinct domains. The redox activity is associated with the Cys65 residue located in N-terminal domain [311], which contains the NLS region, while the enzymatic activity on the abasic sites of DNA is associated with the C-terminal domain [312] (Fig. 8C). REF-1 induces the DNA binding activity of several TFs including AP-1 [307], ATF/CREB family [307], MYB [307], EGR-1 [313], HIF-1α [314], NF–Y [315], PEBP2 [316], TP53 [317], HLF [318], NF-κB [319], and PAX proteins [320] by catalyzing the reduction of cysteine residues of those TFs.

Thioredoxin also plays a role in keeping critical Cys residues in TFs reduced. In fact, overexpression of thioredoxin reverses the oxidation of the Cys62 residue in p50 NF-κB subunit [321], and endogenous thioredoxin is responsible for the maintenance of reducing conditions within the nucleus, so that NF-κB is able to bind to DNA [322]. Furthermore, mutating Cys35Ser in nuclear thioredoxin-1 inhibits NRF2 transcriptional activity and DNA binding [323], which requires reduction of NRF2 Cys506 residue present in the DNA binding domain [298]. Alternatively, thioredoxin may also work as a REF-1 partner because it reduces the oxidized Cys65 of REF-1 [324].

Finally, cysteine oxidation allows discriminating between target sequences in distinct genes. Oxidation of Cys277 in TP53 caused a decrease in TP53 binding to the GADD45, involved in DNA-repair, but not to the p21^(WAF1/CIP1)^ responding element [325].

#### Modulation of DNA binding by PTM

TP53 activation is the most representative example of how a complex network of PTMs, namely phosphorylation, acetylation, ADP-ribosylation, ubiquitylation, sumoylation and neddylation [195,[326], [327], [328], [329], [330]] modulate binding to DNA and TF activity. Most of these modifications occur in the N- and C-terminal transactivation and regulatory domains of TP53, respectively. From the vast information on the literature it seems that the ability of TP53 to specifically select a target gene is closely related to the distinct combinations between PTMs of the TP53 at N-terminal and C-terminal domains like phosphorylation and acetylation, which allows cells to cope and respond to diverse cellular stresses [327,330,332]. H_2_O_2_ modulation of these PTMs further adds a layer of complexity and can contribute for selective gene expression by H_2_O_2_.

##### Phosphorylation of the TF

Concerning TP53, upon stresses the majority of the available sites are rapidly phosphorylated, although some sites (*e.g.* Thr55, Ser376 and Ser378) are constitutively phosphorylated in cells and dephosphorylated in response to stress conditions [327,329]. TP53 can be modified by phosphorylation by a broad range of kinases, including ATM/ATR/DNAPK, and CHK1/CHK2 [329,333]. Concerning exposure of cells to H_2_O_2_ it seems that H_2_O_2_ is able to induce different pathways that lead to diverse TP53 phosphorylation patterns, which is most probably related with used dosages and cell types as summarized in Table 2, but in all the cases leads to transcription of TP53-target genes. In most cases ATM activity is required for these phosphorylations either directly [334,335] or indirectly by other ATM-activated kinases, like PLK3 (Polo-like kinase-3) [335].

Three examples in which phosphorylation of the TF inhibits DNA binding and TF activation are HSF1, SP1 and c-JUN. Concerning HSF1 and SP1 phosphorylation, the kinase involved is JNK [336,337]. For c-JUN, phosphorylation in its C-terminal DNA-binding domain at Thr239 is catalyzed by GSK-3β, while phosphorylation at Ser243 is catalyzed by an unknown protein kinase [338]. This is relevant since the phosphorylation of c-JUN catalyzed by GSK-3 contributes to the repression of growth factor-inducible genes in quiescent cells [339].

Finally, concerning CREB, this TF is regulated *via* phosphorylation at the Ser133 residue present in the P-box of the activation domain. This PTM is attributed to different kinases, depending upon the cell type or the stimulus [95]. H_2_O_2_ can either increase or decrease binding, depending on the cell type. For example, H_2_O_2_ decreases the DNA-binding activity of CREB to the CRE domain in neuronal cells, contrary to what happens in glia cells, where H_2_O_2_ increases the DNA-binding activity of CREB [340]. In immortalized hippocampal neuronal cells (H19-7 cells), a CREB-dependent decrease of both Bcl-2 protein and mRNA levels follows exposure to extracellular H_2_O_2_ (150 µM) [341]. These observations are accompanied by a decrease of CREB phosphorylation. When Bcl-2 is overexpressed in H19-7 cells, these cells are more resistant to apoptosis induced by reactive oxygen species. The loss of CREB function, which is essential for neuronal survival, contributes to oxidative stress-induced neuronal dysfunction. It is predicted that a low level of CREB induced by H_2_O_2_ may influence the apoptosis responses in cells.

##### Acetylation of the TF

In general, H_2_O_2_ increases acetylation of TP53 [342] (see Table 2) and NRF2 [343], and in both cases this correlates with binding to DNA and TF activity. As in the case of NRF2 [343], in most of cases where increased levels of acetylated TP53 induced by H_2_O_2_ were found, a simultaneous decrease in the activity of the deacetylase Sirtuin 1 (SIRT1) also occurs, but the upregulation of target genes can vary in different cell types [344,345]. It should be mentioned that SIRT1 is extremely sensitive to H_2_O_2_ inhibition, since extracellular concentrations as low as 1 μM inhibit SIRT1 by oxidizing critical cysteine residues in the SIRT1 active center [241]. In addition, the RNA-binding protein HUR binds to the 3′ untranslated region of the mRNA encoding SIRT1, leading to its stabilization and increased levels. H_2_O_2_ triggers the dissociation of HUR from the HUR-SIRT1 mRNA complex, promoting SIRT1 mRNA decay, reducing SIRT1 abundance, a process that seems to be regulated by Chk2 kinase [167]. Other TFs also targeted by SIRT1 with the concomitant inhibition of their transcriptional activity are c-JUN, c-FOS and HIF-1α. It is thus tempting to hypothesize that acetylation of these TFs is modulated by H_2_O_2_. SIRT1 may function as a termination pathway because REF-1 was found to reduce SIRT1 cysteine residues, stimulating its activity [241].

Other PTMs compete with acetylation for C-terminal TP53 lysine residues like ubiquitination, methylation, sumoylation, and neddylation [327], which suggests a cross-talk between the different modifications at each specific modifiable residue creating the possibility of a wide variety of patterns of distinct cellular responses to different signals. How H_2_O_2_ affects these PTMs is still unknown but H_2_O_2_ may regulate either the enzymes involved in their addition or removal. Actually, H_2_O_2_ has been implicated as a key regulator of the sumoylation–desumoylation equilibrium by affecting the SUMO conjugating enzymes E1 and E2 [201,202] and SUMO proteases [346].

#### Binding to a co-activator

Binding to a co-activator is a common mechanism of regulation. Regulation at this level by H_2_O_2_ is illustrated by the hydroxylation of HIF. HIF-1α and HIF-2α contain a conserved asparagyl residue that is hydroxylated by factor inhibiting HIF (FIH) enzymes (Asn803 in HIF1α; Asn851 in HIF-2α for human proteins). This hydroxylation inhibits the association with p300, a transcriptional co-activator. It has been shown that FIH is more sensitive to inhibition by H_2_O_2_ than PHD and actually FIH may represent the preferential target for H_2_O_2_-dependent regulation of HIF [347]. In the case of NRF2, mutations at Cys119, Cys235, and Cys506 prevent binding of NRF2 to p300 [348].

#### Release from inhibitor

c-JUN provides a very interesting example in which H_2_O_2_ control is done not by promoting the binding of the TF with a co-activator, but instead it promotes the release of the TF from an inhibitor. Induction of c-JUN is mostly dependent on its upstream kinase, JNK. Once activated, JNK translocates to the nucleus [349] where it catalyzes the phosphorylation of c-JUN at Ser63, Ser73, Thr91 and Thr93 [338,350], and regulates its transcriptional capacity by dissociating c-JUN from a repressor complex that contains HDAC3 [351]. After release from this complex, gene activation, which involves interactions with transcriptional co-activators like histone acetyl transferases, CREB, Smad3 and CBP, can occur in the absence of functional JNK [351] (Fig. 8D). H_2_O_2_ is at the center of these events because JNK is activated by ASK1 [352], and ASK1 is extremely sensitive to H_2_O_2_ by several mechanisms. ASK1 contains 5–6 cysteine residues that, upon exposure to H_2_O_2_, form intermolecular disulfide bonds that mediate ASK1 homo-oligomerization and kinase activity [353]. Thioredoxin-1 reduces these disulfide bonds inhibiting the activation of ASK1. ASK1 is probably not a direct sensor of H_2_O_2_, with its oxidation being mediated by Prx1, which acts as the direct sensor of H_2_O_2_ [29]. An alternative mechanism states that thioredoxin-1 forms an inactive complex with ASK1; upon exposure to H_2_O_2_, thioredoxin is oxidized dissociating the complex, allowing for ASK1 activation [354]. Thioredoxin may be recycled by interaction with REF-1 [324]. Similarly, dissociation of the GST (glutathione S-transferase)-ASK1 complex following GSTp oligomerization promoted by H_2_O_2_ has been proposed to mediate JNK activation [355]. Following oxidation, ASK1 is activated by phosphorylation [353]. Thus, inhibition of serine/threonine protein phosphatase 5 (PP5), an ASK1 phosphatase, has been also proposed as a mechanism for ASK1 activation by H_2_O_2_ [356]. Besides the ASK1/JNK axis, H_2_O_2_ causes oxidation and inhibition of JNK-inactivating phosphatases by oxidizing their catalytic cysteine, thus contributing to JNK activation [357,358].

Another example of TF activation by release from an inhibitor is provided by the Hsf1 yeast counterpart of HSF1. Activation of Hsf1 by H_2_O_2_ in yeast does not involve, apparently, a direct reaction of H_2_O_2_ with the TF, since it exists as a trimer in the nucleus already bound to DNA. Instead, recent evidence suggests that H_2_O_2_ may react with the Hsp70 chaperone Ssa1, which interacts with the transactivation domain of human HSF1 repressing it in the absence of stress [[148], [149], [150],^359^]. In fact, deletion of *SSA1* and *SSA2* genes, which code for the two cytoplasmic isoforms of Hsp70 in *S. cerevisiae*, leads to derepression of Hsf1 transcriptional activity [360]. Wang et al. [359] found that activation of Hsf1 in yeast by H_2_O_2_ probably involves as a direct target the oxidation of two cysteine residues in Ssa1. In this study H_2_O_2_ oxidation of Ssa1 was mimicked by substituting Cys264 and Cys303 with aspartic acid, which resembles a cysteine sulfinic acid, and this led to a non-functional Ssa1 and to constitutive Hsf1 activation.

#### Chromatin state

An illustration on how H_2_O_2_ can affect the state of chromatin locally near the promoter region of specific genes is exemplified by the methylation state of SP1. Recently, SP1 was reported to be specifically methylated *in vivo*, which suppresses its transcriptional activity [361]. In fact, H_2_O_2_ treatments increased the methylation of SP1 and enhanced the interaction between SP1 and HDAC1 in a dose- and time-dependent manner. The experiments were done with HeLa cells treated with 400 µM H_2_O_2_ until 6 h or with 1 mM H_2_O_2_ for 2 h. The negative role induced by the methylation of SP1 is a result of the direct interaction with the protein lysine methyltransferase Suv39H1 (Su(var) 3–9 homologue 1), which allows SP1 to recruit HDAC1 to the promoter region of its target genes. After SP1 methylation associated with Suv39H1 and HDAC1, the chromatin is remodeled with histone deacetylation and histone methylation, culminating in repression of SP1 target genes expression. The authors suggest that the recruitment of HDAC1 is essential for SP1 methylation; however, the enzyme that provides the methyltransferase activity and the residue(s) that are methylated are still unknown [361].

Selective gene expression of individual genes whose promoter regions are exposed may also be accomplished by H_2_O_2_ based on the affinity of promoter regions towards the TF. In the case of NF-κB, simple association–dissociation equilibriums helps to understand why genes with lower affinity towards NF-κB are preferentially modulated by H_2_O_2_ rather than those with higher affinity [164]. Understanding how H_2_O_2_ not just regulates a TF, but also how it selectively discriminates between genes responding to the same TF is essential to have a complete view of regulation of gene expression by H_2_O_2_.

## Conclusions

The complexity of the regulation of TFs by H_2_O_2_ increases from bacteria to unicellular eukaryotes, and from these to multicellular organisms. One consequence is that while the regulatory mechanisms in bacteria and yeast are well defined, a lot of uncertainties still remain in mammalian cells. As noted in a recent review, while in lower organisms a few publications were sufficient to establish the regulatory pathways, in mammalian systems thousands of publications have failed to do so [22]. One example that illustrates the impact of this higher regulatory complexity is NRF2. In an excellent work, the cysteine residues of KEAP1 oxidized by H_2_O_2_ were identified [189]. If NRF2 was to be activated just by a simple oxidation of KEAP1, then the regulatory pathway would be solved. Instead, NRF2 is also regulated by synthesis *de novo*, DNA binding and transactivation and nuclear cytoplasm trafficking. To overcome the problems caused by the higher complexity of mammalian systems, experimental approaches with a more solid quantitative basis are needed. The concept of operating mechanism should be in place. When a new mechanism is discovered, it is imperative to know if it is operating *in vivo* and to assess its relative importance compared with alternative competing pathways. Otherwise the newly discovered mechanism will certainly add new information but its biological meaning is uncertain. Here we deduce a set of simple equations that help to extract kinetic quantitative data from typical experiments. Although, these equations are simplifications and may lack the rigor of a true kinetic approach, they, hopefully, can be easily applied to typical experimental data present in most signaling papers. We exemplified this with a couple of calculations arriving to a conclusion that in HeLa cells KEAP1 reactivity with H_2_O_2_ is weak, while a highly reactive sensor is probably mediating stimulation of NRF2 synthesis *de novo* by H_2_O_2_.

Part of the complexity mentioned above may be artificial because there is a tendency to integrate information coming from different types of cells. One of the consequences of multicellularity is cell specialization and so different signaling mechanisms operate in different cells. For example, H_2_O_2_ activates SP1 translation only in neurons and not in glia cells. In spite of this cellular specialization, it is tempting to identify a set of players that recurrently appear as intermediates in H_2_O_2_ signaling pathways and that may function as hubs, namely ASK1/MAPK; GSK3β, ATM, c-ABL, SIRT1, REF-1, as well as several phosphatases. Many of these molecules are kinases and phosphatases, underlying the well-known fact that H_2_O_2_ affects the phosphorylation state of signaling pathways. Recent advances with the discovery of the inhibition of Prxs by phosphorylation, show that this is a two-way relationship where signaling by phosphorylation/dephosphorylation affect the levels of H_2_O_2_ by decreasing its removal. If other thiol proteins have their SH groups reactivity with H_2_O_2_ also affected by its phosphorylation state, a cross-talk between protein thiol oxidation state and protein phosphorylation state would be established.

Our calculations shown in Table 1 help to understand how H_2_O_2_ signaling can be specific. Depending on the time of exposure and on H_2_O_2_ concentration different sensors may be activated. This chemical specificity combined with the localized production of H_2_O_2_, where only a handful of molecular sensors are available, yields a high degree of biological specificity. We have proposed that H_2_O_2_ is not only a generic modulator of TFs and signaling molecules, but has the potential of being a specific regulator of individual genes [115], and the vast array of regulatory mechanisms by which H_2_O_2_ exerts its effects described here supports such view. To know which genes are activated by H_2_O_2_ in each type of tissue and how this is affected by the presence of single nucleotide polymorphisms in the promoter regions of genes is essential to have a complete view of the regulation of gene expression by H_2_O_2_. Ultimately, such understanding will be essential for redox biology to be an active player in the emerging area of personalized medicine.
